# Supplementary material for: What are the experiences of teleophthalmology in optometric referral pathways? A qualitative interview study with patients and clinicians
Source: BMJ Open. 2024 May 24;14(5):e078161. doi: 10.1136/bmjopen-2023-078161 (PMC11129051; doi:10.1136/bmjopen-2023-078161)
Supplement: Supplementary data [file bmjopen-2023-078161supp001.pdf]

Interview topic guide- Patients

1. Information and consent

- Briefly re-introduce the topic, the purpose of the overall project and the specific aim of the HCI study
- Provide assurances regarding confidentiality and how information will be processed
- Check willingness to continue to participate
- Obtain informed consent (if not taken in advance) and permission to be audio/video recorded

2. Introduction

Thank you for taking part in this interview. We really appreciate you taking the time to talk with us. As I explained earlier, we are keen to talk to you about the use of new tele-eye care technologies in the referral process between community optometric practices and hospital eye services. We want to hear your views on these technologies and whether they would work for you in either a good or bad way. There are no right or wrong answers, so feel free to tell us what you think, and if there is something that needs to be improved.

The interview is likely to take around 30 minutes, you are free to stop and take a break whenever you feel like. It is also voluntary, which means you can opt out at any time for any reason.

3. Background information

- So, first of all: I would like to know more about you and your use of primary and secondary eye services.
- How old are you?
- Do you have any known eye problems?
- How often do you visit your optician?
  - Do you do regular check-ups, or do you visit the practice when you have eye problems only?
  - When was your last visit to optometric practice [i.e., the one before your most recent appointment)?

- What led to you to making your most recent eye appointment [i.e., the one that has led to the referral]?
- What did you expect to happen during that particular appointment?

- Have you been referred to hospital for eye care before?

4. Questions related to the teleophthalmology platform

- Can you describe to me your most recent visit to the optometric practice services? [i.e., the one you just had with the OCT scan?]
  - Can you describe to me the steps that you went through during the consultation with the optometrist?
  - **For the intervention arm:**
    - Have you noticed any difference between the care you received this time and last time? Were you asked to do something different during this consultation to what you were used to do? What was it? What are your thoughts on this?
  - Were the OCT scan results discussed with you? When were you informed about the referral decision?
  - What were you told will happen next? How did you feel about that?
  - How long did you tell you it will take to see a doctor?
- **For the intervention arm**
- Have you **received feedback** about your referral?
  - How long did it take from the time you were referred by your optician to the time you received the feedback (referral letter)? What do you think about this waiting time?
  - What were the information included in the feedback letter? (do you remember?), what do you think about that?
  - What happened next?
- **For the control arm**
    - What do you think a tele-eye care service or a teleophthalmology service in the referral process is? **Explain what teleophthalmology is in the context of the HERMES project.**
- Generally, what do you think about this new referral process?
- What do you think the benefits of this service are?
- What do you think the problems with this service are?

- What do you think the disadvantages would be for you as a patient if this type of service was implemented in real life?
- In this type of service, an optician at the community optometry practice and an ophthalmologist at the hospital will be involved in the referral process, who would you be most confident in their decision? And why?
- How would you feel if you were referred to hospital for eye care when it was not necessary?
    - How would you feel spending this time on a visit that wasn't necessary?
    - What do you think should be done to avoid referring you when it is not necessary?
- Some of the opticians refer all cases with suspected retinal conditions to hospital, which may include those who are urgent and those that turn out to be unnecessary (unnecessary referrals). So when hospital receive these referrals, the hospital has two options- **Option 1:** See all patients referred by opticians and provide re-assurances to everyone regarding their eye conditions or **Option 2:** hospital sees mainly urgent cases who require hospital care.
    - What do you think of these options? Which one would you prefer? Why?

6. End of the interview

We have reached the end of the interview. Would you like to share any more ideas or thoughts?

Thank you so much for taking the time to participate in the study, we will use this information to inform the design of a future tele-eye care service.

Interview topic guide – Primary care professionals - Control

1. Information and consent

- Briefly re-introduce the topic, the purpose of the overall project and the specific aim of the HCI study
- Obtain informed consent (if not taken in advance) and permission to be audio/video recorded

2. Introduction

Thank you for taking part in this interview. We really appreciate you taking the time to talk with us. As I explained earlier, we are keen to talk to you about the use of new teleophthalmology technologies in the referral process between community optometry practices and hospital eye services. We want to hear your views on these technologies, and what factors you think may influence their implementation in real life**.** There are no right or wrong answers, so feel free to tell us what you think, and if there is something that needs to be improved.

The interview is likely to take around 30-45 minutes. Your participation is voluntary, and you can withdraw from the study at any time.

3. Background Information

- First of all: I will start by asking you a few questions to know more about you.
- What is your professional background?
- How long have you been working in community optometry practices?
- How often do you refer patients with retinal conditions to hospital eye services? (in a week what is the average number of cases identified with retinal conditions)
- Can you explain to me the different referral pathways that you follow when you identify someone with a suspected retinal condition (usual not through the teleophthalmology platform)
- What do you think of the referral process?
    - What are the issues or barriers that you face when referring patients?
- How do you think the referral process can be improved?

4. Teleophthalmology platform

4.1 Expectation/coherence

- What do you think a teleophthalmology platform is? How different is it from the traditional system?
- What would you like to see in a teleophthalmology platform?
- How do you think a teleophthalmology platform would benefit/improve the referral process for eye conditions?

- What barriers do you think there will be to using a teleophthalmology platform in the context of referrals?
- What do you think would need to change to incorporate a teleophthalmology platform in your practice?

6. End of the interview

We have reached the end of the interview. Would you like to share any more ideas or thoughts?
Thank you so much for taking the time to participate in the study, we will use this information to inform the design of future teleophthalmology services.

Interview topic guide – Primary care professionals - Intervention

1. Information and consent

- o Briefly re-introduce the topic, the purpose of the overall project and the specific aim of the HCI study
- o Obtain informed consent (if not taken in advance) and permission to be audio/video recorded

2. Introduction

Thank you for taking part in this interview. We really appreciate you taking the time to talk with us. As I explained earlier, we are keen to talk to you about the use of new teleophthalmology technologies in the referral process between community optometry practices and hospital eye services. We want to hear your views on these technologies, and what factors you think may influence their implementation in real life. There are no right or wrong answers, so feel free to tell us what you think, and if there is something that needs to be improved.

The interview is likely to take around 30-45 minutes. Your participation is voluntary, and you can withdraw from the study at any time.

3. Background Information

- First of all: I will start by asking you a few questions to know more about you.
- What is your professional background?
- How long have you been working in community optometry practices?
- How often do you refer patients with retinal conditions to hospital eye services? (in a week what is the average number of cases identified with retinal conditions)
- Can you explain to me the different referral pathways that you follow when you identify someone with a suspected retinal condition (usual not through the teleophthalmology platform)
- What do you think of the referral process?
    - o What are the issues or barriers that you face when referring patients?
- How do you think the referral process can be improved?

4. Teleophthalmology platform

4.1 Experience/cognitive participation

- How long have you been using this new teleophthalmology platform?
- Can you describe to me the steps you follow when you refer a patient with a suspected retinal condition through the new teleophthalmology platform?
- How long does it take you to do a referral on this platform? What do you think of that?
- What did you like about this new platform? Why?
- What did you not like about this new platform? Why?
    - o What were the issues that you faced when using the platform?
    - o Do you have any concerns regarding the use of this platform?
- What type of support did you receive to use this platform (e.g., training, support staff)?

4.2 Experience/collective action

- What changes did you have to make in your work routine to incorporate this new technology?
- How will this new platform fit in your current practice?
- How do you think this new platform will impact/improve your practice, patient care and your working relationship with secondary care?

4.3 Reflective monitoring

- What do you think will be the benefits of this new teleophthalmology platform on the referral process, both short term and long term?
  - How do you think this new teleophthalmology platform can help reduce unnecessary referrals?
  - How do you think this new teleophthalmology platform can help improve the quality of referrals?
- What barriers/disadvantages do you think there will be to using this new platform on the referral process?
- What do you think would need to change for this technology to be successfully embedded in routine eye practice (e.g., training…)?

6. End of the interview

We have reached the end of the interview. Would you like to share any more ideas or thoughts?

Thank you so much for taking the time to participate in the study, we will use this information to inform the design of future teleophthalmology services.

<u>Questions for ophthalmologists:</u>

- I will be asking you a few questions regarding the use of the new teleophthalmology platform in the referral process.
- I will audio record the session, are you happy for me to do so? Consent form points.
  1. Can you sketch out the overall referral process for retinal conditions and how it works from your trust’s perspective?
  2. What are the main issues you face in the referral process from community optometry practices?
  3. About how many new retinal referrals do you deal with per week? Usually how do you organize this aspect of your work? ** Usual practice**
  4. For the HERMES platform, how are you notified about new referrals on the system? How often do you check the system for new referrals, or how soon do you review the referral? How many referrals do you receive in this system per week?
  5. How long does it take you to review each referral? What do you think of this duration? Reasonable, good, long, can be improved?
  6. Who else check the referrals on the teleophthalmology platform at your trust? How do you coordinate your work with them? Do you distribute the referrals amongst

yourselves, or do you review each new referral? How do you validate your referral decision?

7. So far, what do you think about the quality of the referrals received by this platform? Have you observed any changes compared to usual route?
8. What are the things that you liked about the platform, things that are working well so far?
9. What are the pain points of the platform? What are the things that you did not like? And how can they be improved?
10. What happens next, after you decided on the referral?
11. Looking to the future, how do you envision that this system will be rolled out across your trust or are a couple of consultants always going to be able to assess all OCT scans?
12. What do you think would need to change for this technology to be successfully embedded in routine eye practice?
