# Supplementary material for: What are the experiences of teleophthalmology in optometric referral pathways? A qualitative interview study with patients and clinicians
Source: BMJ Open. 2024 May 24;14(5):e078161. doi: 10.1136/bmjopen-2023-078161 (PMC11129051; doi:10.1136/bmjopen-2023-078161)
Supplement: Author's manuscript [file bmjopen-2023-078161.draft_revisions.pdf]

BMJ Open

BMJ Open is committed to open peer review. As part of this commitment we make the peer review history of every article we publish publicly available.

When an article is published we post the peer reviewers' comments and the authors' responses online. We also post the versions of the paper that were used during peer review. These are the versions that the peer review comments apply to.

The versions of the paper that follow are the versions that were submitted during the peer review process. They are not the versions of record or the final published versions. They should not be cited or distributed as the published version of this manuscript.

BMJ Open is an open access journal and the full, final, typeset and author-corrected version of record of the manuscript is available on our site with no access controls, subscription charges or pay-per-view fees (http://bmjopen.bmj.com).

If you have any questions on BMJ Open's open peer review process please email info.bmjopen@bmj.com

BMJ Open

Experiences of Teleophthalmology in Optometric Referral Pathways: a qualitative interview study

Journal:	*BMJ Open*
Manuscript ID	bmjopen-2023-078161
Article Type:	Original research
Date Submitted by the Author:	25-Jul-2023
Complete List of Authors:	Patel , Dilisha ; University College London, UCL Interaction Centre<br>Abdi, Sarah; University College London, UCL Interaction Centre<br>Carmichael, Josie; University College London, UCL Interaction Centre<br>Balaskas, Konstantinos; Moorfields Eye Hospital NHS Foundation Trust,<br>Blandford, Ann; University College London, UCL Interaction Centre
Keywords:	Telemedicine < BIOTECHNOLOGY & BIOINFORMATICS, Public health < INFECTIOUS DISEASES, OPHTHALMOLOGY

SCHOLARONE™
Manuscripts

I, the Submitting Author has the right to grant and does grant on behalf of all authors of the Work (as defined in the below author licence), an exclusive licence and/or a non-exclusive licence for contributions from authors who are: i) UK Crown employees; ii) where BMJ has agreed a CC-BY licence shall apply, and/or iii) in accordance with the terms applicable for US Federal Government officers or employees acting as part of their official duties; on a worldwide, perpetual, irrevocable, royalty-free basis to BMJ Publishing Group Ltd ("BMJ") its licensees and where the relevant Journal is co-owned by BMJ to the co-owners of the Journal, to publish the Work in this journal and any other BMJ products and to exploit all rights, as set out in our licence.

The Submitting Author accepts and understands that any supply made under these terms is made by BMJ to the Submitting Author unless you are acting as an employee on behalf of your employer or a postgraduate student of an affiliated institution which is paying any applicable article publishing charge ("APC") for Open Access articles. Where the Submitting Author wishes to make the Work available on an Open Access basis (and intends to pay the relevant APC), the terms of reuse of such Open Access shall be governed by a Creative Commons licence – details of these licences and which Creative Commons licence will apply to this Work are set out in our licence referred to above.

Other than as permitted in any relevant BMJ Author's Self Archiving Policies, I confirm this Work has not been accepted for publication elsewhere, is not being considered for publication elsewhere and does not duplicate material already published. I confirm all authors consent to publication of this Work and authorise the granting of this licence.

For peer review only

Experiences of Teleophthalmology in Optometric Referral Pathways: a qualitative interview study

Authors Anon

What is already known on this topic - There are immense pressures on hospital eye services, with a backlog of patients. Many patients waiting to be seen are unnecessary referrals; however, this is not identified until they attend clinic. Currently, many community-based optometrists are not routinely involved in the referral pathway and feel undervalued and uninformed about their patient's care.

What this study adds - There is potential value in implementing teleophthalmology into the optometric referral pathway, but little is known about how this would work in practice. We present the novel lived experiences of patients, optometrists, and ophthalmologists of teleophthalmology in practice (implemented in the context of a clinical trial). We identify key considerations that are needed to support successful implementation.

Synopsis (31 words)- We present patients', optometrists', and ophthalmologists' experiences of teleophthalmology in the optometric referral pathway. Teleophthalmology was welcomed as improving efficiency by triaging referrals and improving patient care by providing referral feedback.

Abstract

OBJECTIVE: Implementing teleophthalmology into the optometric referral pathway may ease the current pressures on hospital eye services caused by over-referrals from optometrists. This study aimed to understand the practical implications of implementing teleophthalmology by analysing lived experiences and perceptions of teleophthalmology in the optometric referral pathway for suspected retinal conditions.

DESIGN/PARTICIPANTS: We interviewed 41 participants: patients (17), optometrists (18), and ophthalmologists (6). Through thematic analysis, we collated and present their experiences of implementing teleophthalmology.

RESULTS: All participants interviewed were positive towards teleophthalmology as it could enable efficiencies in the referral pathway and improve feedback and communication between patients and healthcare professionals. Concerns included setup costs for optometrists and anxieties from patients about not seeing an ophthalmologist face-to-face. However, reducing unnecessary referrals and increasing the availability of resources and capacity were seen as significant benefits.

CONCLUSIONS: Overall, we report positive experiences of implementing teleophthalmology into the optometric referral pathway for suspected retinal conditions. Successful implementation will require appropriate investment to set up and integrate new technology and remunerate services, and continued evaluation to ensure feedback to patients and between healthcare professionals is received.

Strengths and Limitations:

- The strengths of our study include our ability to report on participants' rich and compelling lived experiences through in-depth qualitative interviews of using and being involved in referral pathways that used teleophthalmology over prospective opinions about the technology.
- We can further qualify previous literature that has reported teleophthalmology's potential impacts and benefits with these lived experiences within the HERMES study.
- This study collected data from multiple stakeholders, including primary and secondary eye care professionals and patients.
- A limitation of this study was that all ophthalmologists interviewed were involved in the HERMES study and, therefore may have had more knowledge of teleophthalmology than others.

For peer review only

Introduction

Primary eyecare in the U.K. is mainly delivered by community optometry practices [1]. If patients are suspected of having a retinal condition, referrals to hospital eye services (HES) are typically processed by their general practitioner (G.P.) based on recommendations from the community-based optometrist. Thus, optometrists are often not involved in the actual referral pathway or informed of outcomes. The additional step can reduce the specificity of clinical details included in the referral, as GPs are not specialists in eye care and rarely have the time or expertise to undertake eye examinations [2]. For several reasons, including concerns over capacity, changes in practice due to the Covid-19 pandemic, and the high number of referrals to HES, there is a need for disruptive changes in the optometric referral pathways for suspected retinal conditions (SRC) [3]. We explore the experiences of patients, optometrists, and ophthalmologists of teleophthalmology for SRC.

Teleophthalmology describes providing health information with medical technology at a distance, geographical, temporal or both [4, 5], to facilitate decision-making. In our study, we used an asynchronous platform; this allowed patients to be referred by uploading clinical information and ocular scans directly from the optometry practice for later review by the receiving HES-based ophthalmologists. A benefit of the custom-built study platform, when compared to recent attempts for the commissioning of teleophthalmology-like store-and-forward referral pathways, is that it enables optometrists to upload full-volume optical coherence tomography (OCT) scans from multiple device manufacturers, in both their proprietary and open-source file format, which can then be parsed and reconstructed into a high-quality full volume scan, directly viewable on the platform's embedded viewer [6]. Given the critical role of OCT imaging for diagnosing and managing medical retina conditions, a minimum clinical dataset including full-volume OCT is a prerequisite for the safe and efficient delivery of teleophthalmology referral triaging, providing ophthalmologists with essential information to confidently make referral triaging decisions. In the absence of teleophthalmology, or when attempts to implement teleophthalmology-like pathways are made with minimal input from clinical informatics and HES-based clinician and imaging experts, triaging ophthalmologists receive referrals, either without any accompanying imaging or frequently with one or a few selected cross-sectional images, not the entire volume [7], then referred patients are seen face to face to assess whether further investigation or treatment is needed. The non-urgent referral pathway with and without teleophthalmology is presented in Figure 1. A recent systematic review reported that teleophthalmology and digital referrals could reduce waiting time, costs, and unnecessary referrals. It also noted that teleophthalmology could lead to earlier detection and diagnosis [8]. This review was based on reviewing referrals, not people's first-hand experiences. Therefore, there is a need to understand how implementation would affect users in practice.

Reports have shown that the growing use of OCT scanning has increased the number of referrals to HES [9, 10]; therefore, teleophthalmology has been suggested to reduce unnecessary referrals, manage growing capacity concerns and potentially manage increasing workloads by reviewing referrals before patients are seen in clinics.

We present findings from a study linked with a cluster randomised clinical trial (HERMES) evaluating the effectiveness of a teleophthalmology platform [1]. Those in the intervention arm of the trial were using teleophthalmology to refer patients to HES, while those in the control arm were using their regular referral pathways. We report the novel qualitative findings of patients' and healthcare professionals' experiences to understand the practical implications of implementing teleophthalmology.

Materials and Method

This study's overall aims, objectives and recruitment strategy are detailed elsewhere [11]; we summarise key points here. We undertook semi-structured interviews with 41 participants between December 2021 and May 2022. These comprised 6 ophthalmologists who were all participating in the study and making remote referral decisions; 18 community optometrists, of whom 12 were from the intervention arm and 6 from the control arm of the HERMES study; and 17 patients recruited from community practices, of which 14 were from clinics in the intervention arm and 3 from clinics in the control arm. A semi-structured topic guide was used for all interviews and was tailored to each participant group. All interviews were audio-recorded with consent. Five interviews were manually transcribed verbatim to aid familiarisation; the remaining interviews were transcribed using Scrintal Software. All transcripts

were anonymised and checked for accuracy. The transcripts were coded using NVivo by an independent researcher who did not conduct the interviews, using inductive thematic analysis methods [12, 13]. 35 codes were initially defined and discussed with the research team. These were then refined and categorised into overarching themes. We present the three main themes which explore experiences of teleophthalmology for referrals for SRC.

Ethics Statement

Ethical approvals were gained from London-Bromley Research Ethics Committee (REC 20/LO/1299). Informed consent was obtained from participants after details of the study, including the aims and methods, were disclosed.

Results

We present our findings under three themes, *Efficiencies of Teleophthalmology; Teleophthalmology enables Feedback* and *Concerns about Teleophthalmology.* We found most participants were optimistic about the implementation of teleophthalmology in the optometric referral pathway due to the efficiencies the platform would enable. All participants expressed needing feedback during the referral process to improve care and highlighted some concerns.

Efficiencies of Teleophthalmology

All welcomed Teleophthalmology due to its ability to improve patient and clinician experiences. There is regional variation in referral pathways depending on the specific condition; GPs typically process routine referrals for SRC. However, it was reported that GPs were not always suited to create referrals due to their limited skillset in specialist eyecare. Patients often described GPs as the unnecessary "middleman". Patients reported being keen to be referred directly by their optometrist and felt this would reduce their waiting time to hear back from HES if referrals were processed directly.

"If the optician can do the referral directly rather than you know, the optician telling me you'll need to go and see your GP who will refer you (...) I would be more comfortable because they [optometrist] know what they're doing whereas the GP is just saying you are alright then, if your optician told you that, then I'll send you." (Patient 11)

In the HERMES study, optometrists used teleophthalmology to refer patients to HES, enabling a quicker referral process direct to triaging ophthalmologists. They shared that teleophthalmology could improve patient satisfaction and help relieve hospital capacity pressures by reducing unnecessary hospital visits.

"I think the whole teleophthalmology thing will improve patient satisfaction and it makes life a lot easier, less patients, elderly patients having to find transport to the hospital, and being dilated once in the optometry practice and then being dilated again, back at the hospital and patient transport having to be arranged, so overall good, big saving of cost and finance and less crowded waiting rooms at the hospital." (Optometrist 18)

Ophthalmologists shared that the teleophthalmology platform introduced uniformity to the referral process by requiring the same data fields to be completed for each patient, which was easy for the referring optometrist. This enabled referrals to be triaged and reviewed more quickly: referral decisions could be made promptly, and the appropriate triaging decision could be made regarding the indication and the urgency for a hospital visit.

"It's more informative because, as you know, the platform has the questions with the tick box, um, on the optician findings which [is] not always involved in a classic referral proforma. And obviously, it has the imaging as well, which helps us to make a decision very quickly." (Ophthalmologist 1)

As mentioned, the key benefit of the teleophthalmology platform is the ability to review and triage patient referrals without them having to attend a face-to-face appointment. All participants recognised and shared the advantage of saving time and resources by using teleophthalmology.

Teleophthalmology Enables Feedback

Some patients reported that they were happy for teleophthalmology to be used to assess their referrals as they would not want to attend HES if not required. Still, they expected feedback to explain the reason for non-referrals. This was not always provided. Some patients reported dissatisfaction with their referral experience due to the lack of communication. This was also shared in the context of not receiving feedback promptly. Patients expected to hear about their referral decision more promptly through the teleophthalmology process, which increased concerns over their eye health when this expectation was not satisfied. In these cases, patients wanted to be seen or told directly and promptly why an appointment was not required and not to be kept waiting in uncertainty. Teleophthalmology can overcome this expectation discrepancy through accurate information presentation and timely and clear feedback.

"If the patients were to get a letter or some form of communication from the hospital or the specialist to reassure them that your case has been looked at and this is what has been concluded, I think that would be enough to put somebody's mind at ease." (Patient 10)

Optometrists stated that one of the significant benefits of teleophthalmology was the ability to receive feedback from ophthalmologists. Optometrists reported that when patients were referred to HES in the absence of teleophthalmology, patients would often return to them seeking more information and advice about their care; therefore, it was important for optometrists to be involved in the referral pathway and remain informed of their patients' management plans. Optometrists shared that many patients were not a reliable source of information about their eye health/treatment, which could affect future care or monitoring they provided.

"Once we refer the patient, we don't actually know then what is happening thereafter, unless we chase the patient or, our patients are quite loyal, so they would, we would see them a year later, we will say or remember, we referred you last time, what happened? (…) So, it's actually we're basing it of what the patient is then telling us, so we are actually getting like the second story through the patient rather than the actual clinical information." (Optometrist 12)

By receiving feedback, optometrists can also verify whether their referrals were appropriate and audit themselves to improve the quality of their referrals.

"Because if we keep referring something that we think is urgent, but [the] ophthalmologist tells us this is not urgent, and if you learn by that, that's going to help you, you see. Right now, there's no feedback. (…). But if I got feedback from the ophthalmologist that saw the patient and I will know for next time when I see that similar sort of situation that well, actually this isn't urgent." (Optometrist 10)

Having a system where the community-based clinic is connected to the HES was also seen as a great benefit for ophthalmologists. They welcomed being able to provide feedback to the referring optometrists, especially to enable the sharing of referral decisions directly and concurred with the need to provide feedback on the referral quality to improve future referrals. It was suggested that the teleophthalmology system should send referral replies to the referring optometrist, patient, and GP so all are informed of the outcome.

Concerns about Teleophthalmology

There were some concerns about using technology to manage patient referrals. Some patients still wanted the reassurance of seeing a clinician rather than having their referral decision and notification completed remotely. Seeing someone face-to-face provided the holistic care some patients reported wanting and addressed their worries and anxieties.

"I think [if] you don't get a chance to see the patient yourself, there is something about looking at data visually transactionally, that is fine, but there is also something about talking to the patients about how they're feeling and how they're coping with things." (Patient 10)

For peer review only

Optometrists were mainly concerned with the practicalities of implementing a new system into their workflow. This included concerns over training to use the equipment, the reliability of network connectivity and equipment costs that some smaller practices may not be able to bear, as well as remuneration for their time for taking on additional roles. Some also reported that completing a referral on the teleophthalmology platform took time.

"The barriers would be cost, because this is all based on the information that is being sent from an OCT device, yeah, as part of the process of referral, it's not just from a letter, so when it comes to having the equipment, that's an immediate barrier. And having the right remuneration for the equipment." (Optometrist 2)

Ophthalmologists also shared these concerns; however, they were positive towards the ability of teleophthalmology, enabling them to use their time more efficiently.

Discussion

Whilst previous work has focused on the efficacy and efficiency of teleophthalmology platforms through reviewing referrals [10, 14], we report insights based on experiences from patients, optometrists, and ophthalmologists to validate previous findings on perceptions of using teleophthalmology for suspected retinal conditions.

All participants recognised the value of implementing a teleophthalmology system into their ophthalmic care pathway due to its potential to improve patient care and health services efficiencies. This is supported by others [15], who found through a review of referrals that teleophthalmology can reduce unnecessary referrals and significantly impact patient anxieties [16]. Our study has shown this in practice, with many patients sharing that they would not want to attend HES if not required.

Both optometrists and ophthalmologists reported that teleophthalmology's significant advantage is the ability to electronically refer patients directly from optometrist practices to hospital eye services, which can significantly reduce the waiting time for patients, and they were beginning to see in practice. The ability to triage referrals electronically also enabled ophthalmologists to provide replies and feedback to the referring optometrist via the teleophthalmology platform, which they greatly valued.

Implementing teleophthalmology into the eye care pathway would remove the burden from GPs of having to process patient referrals, but they must be informed of such referrals. GPs have, in principle, supported the suggestion of optometrists referring patients directly [14], and we found that patients and optometrists would support this change in practice. There is great value in involving optometrists in the referral process, as it has been reported that this could reduce unnecessary referrals by approximately half [10].

The overarching theme shared by all participants that substantiate many of the benefits of the teleophthalmology platform is the potential of the platform to facilitate the provision of feedback. The importance of receiving timely feedback in eye care, in general, has been reported by others [2, 17] and was essential for patients to alleviate their concerns over their eye health. Harvey et al. (2022) specifically outlines the factors that could affect the provision of feedback, and a key implication of their work is the call for technology to support this provision. Thus, we found that teleophthalmology could overcome concerns in the optometric referral pathway.

Whilst feedback can keep both patients and optometrists informed [18], it can also improve future referral quality through open conversations between referring clinicians. Our results concur as we report optometrists greatly value receiving referral replies directly from ophthalmologists to remain informed of their patients' care and audit their referrals. Additionally, previous research has highlighted that the lack of communication between optometrists and ophthalmologists can be problematic [19, 20]; therefore, implementing a teleophthalmology platform could help to overcome this.

Potential barriers raised by some optometrists were the initial setup costs, which include time, training, and financial costs. Whilst others have reported this should be considered [8, 14], we found this to be a key concern in practice. Therefore, there is a need to ensure optometrists are appropriately remunerated for providing this service. Others

For peer review only

have begun to explore the cost-effectiveness of implementing teleophthalmology systems [21]; further work is needed to establish health-economic benefits concerning SRC that were raised in our study.

To successfully implement teleophthalmology into the optometric referral pathway, there needs to be an investment to enable parity amongst optometry practices to support new technology setup. Whilst the implementation of teleophthalmology was perceived to initially increase the workload of optometrists to process referrals and ophthalmologists to triage referrals, with the correct remuneration there is significant potential to relieve pressures on stretched eye care services.

Conclusions

Implementing teleophthalmology into the optometric referral pathway has numerous benefits, as outlined by all participants. Through our in-depth interview study with patients, optometrists, and ophthalmologists, we found that they generally report great value in implementing teleophthalmology through improving efficiency and the ability to provide and receive feedback. Patients were satisfied if their referrals were reviewed with teleophthalmology to reduce the possibility of unnecessary HES visits if this was clearly and efficiently communicated back to them. Optometrists felt they were better suited than GPs to write and process patient referrals and would feel more valued if they were more directly involved in the pathway. Finally, ophthalmologists were pleased with a system enabling them to manage their caseloads more efficiently. Further efficiencies teleophthalmology can promote include removing the burden on GPs, the time patients wait to be seen by HES, the time it takes for ophthalmologists to review and provide referral replies and finally, the overarching benefit to all participants of being involved and receiving feedback. Future work could explore the specific health economics of implementing teleophthalmology to validate our findings.

Acknowledgements

We thank all those involved in this research, including participants and the wider research team.

Funding Statement

This work was funded by NIHR Health Technology Assessment grant number 18/182.

Competing Interests

We have no competing interests to declare.

Author Contributions

1-analysed the data and prepared the manuscript. 2,3 &5 designed the study protocol, 2 undertook recruitment and data collection. 2,3& 5 contributed analysis as presented by 1. All authors reviewed and contributed to subsequent drafts and approved the final manuscript.

References

1. Han, J.E.D., et al., Teleophthalmology-enabled and artificial intelligence-ready referral pathway for community optometry referrals of retinal disease (HERMES): a Cluster Randomised Superiority Trial with a linked Diagnostic Accuracy Study-HERMES study report 1-study protocol. BMJ Open, 2022. 12(2): p. e055845.
2. Pierscionek, T., J. Moore, and B. Pierscionek, Referrals to ophthalmology: optometric and general practice comparison. Ophthalmic and Physiological Optics, 2009. 29(1): p. 32-40.
3. Tham, Y.C., et al., New digital models of care in ophthalmology, during and beyond the COVID-19 pandemic. Br J Ophthalmol, 2022. 106(4): p. 452-457.

For peer review only

4. Caffery, L.J., et al., Models of care in tele-ophthalmology: A scoping review. J Telemed Telecare, 2019. 25(2): p. 106-122.
5. Surendran, T.S. and R. Raman, Teleophthalmology in Diabetic Retinopathy. J Diabetes Sci Technol, 2014. 8(2): p. 262-266.
6. Roberts, C. How digital eye care referrals will transform care. NHS England- Transformation Directorate 2021 10/05/2023]; Available from: https://transform.england.nhs.uk/blogs/how-digital-eye-care-referrals-will-transform-care/.
7. Committee, H.a.S.C., Evaluation of Governement commitments made on the digitisation of the NHS. Fourth Special Report of SEssion 2022-23. 2023: https://publications.parliament.uk/pa/cm5803/cmselect/cmhealth/780/report.html
8. Conway, M.P., et al., Investigating the Role of Optometrists in Teleophthalmology and the Implications of Increasing Access to Advanced Imaging Techniques and Digital Referral: A Systematic Search and Review. Telemedicine and e-Health, 2021. 27(9): p. 974-981.
9. Lee, J.X., V. Manjunath, and S.J. Talks, Expanding the role of medical retina virtual clinics using multimodal ultra-widefield and optical coherence tomography imaging. Clin Ophthalmol, 2018. 12: p. 2337-2345.
10. Kern, C., et al., Implementation of a cloud-based referral platform in ophthalmology: making telemedicine services a reality in eye care. Br J Ophthalmol, 2020. 104(3): p. 312-317.
11. Blandford, A., et al., Protocol for a qualitative study to explore acceptability, barriers and facilitators of the implementation of new teleophthalmology technologies between community optometry practices and hospital eye services. BMJ Open, 2022. 12(7): p. e060810.
12. Braun, V. and V. Clarke, Successful qualitative research: A practical guide for beginners. 2013: sage.
13. Braun, V. and V. Clarke, Using thematic analysis in psychology. Qualitative Research in Psychology, 2006. 3(2): p. 77-101.
14. Kelly, S.P., et al., Teleophthalmology with optical coherence tomography imaging in community optometry. Evaluation of a quality improvement for macular patients. Clinical Ophthalmology (Auckland, NZ), 2011. 5: p. 1673.
15. Muttuvelu, D.V., et al., Danish teleophthalmology platform reduces optometry referrals into the national eye care system. BMJ Open Ophthalmology, 2021. 6(1): p. e000671.
16. Davey, C.J., C. Harley, and D.B. Elliott, Levels of state and trait anxiety in patients referred to ophthalmology by primary care clinicians: a cross sectional study. PLoS One, 2013. 8(6): p. e65708.
17. Harvey, K., et al., Referrals from community optometrists in England and their replies: A mixed methods study. Ophthalmic and Physiological Optics, 2022. 42(3): p. 454-470.
18. Baldwin, D.M., et al., Patient preferences for notification of normal laboratory test results: a report from the ASIPS Collaborative. BMC family practice, 2005. 6(1): p. 1-7.
19. O'Malley, A.S. and J.D. Reschovsky, Referral and consultation communication between primary care and specialist physicians: finding common ground. Archives of internal medicine, 2011. 171(1): p. 56-65.
20. Townsend, D., et al., Health professionals' and service users' perspectives of shared care for monitoring wet age-related macular degeneration: a qualitative study alongside the ECHoES trial. BMJ open, 2015. 5(4): p. e007400.
21. Sharafeldin, N., et al., Review of economic evaluations of teleophthalmology as a screening strategy for chronic eye disease in adults. Br J Ophthalmol, 2018. 102(11): p. 1485-1491.

For peer review only

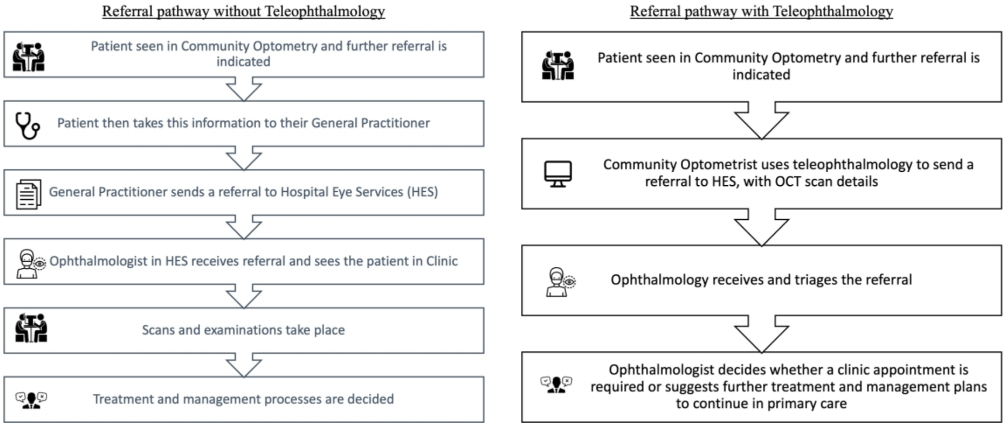

317x135mm (330 x 330 DPI)

Standards for Reporting Qualitative Research (SRQR)*

http://www.equator-network.org/reporting-guidelines/srqr/

Title and abstract

	Page/line no(s).
**Title** - Concise description of the nature and topic of the study Identifying the study as qualitative or indicating the approach (e.g., ethnography, grounded theory) or data collection methods (e.g., interview, focus group) is recommended	1
**Abstract** - Summary of key elements of the study using the abstract format of the intended publication; typically includes background, purpose, methods, results, and conclusions	1

Introduction

**Problem formulation** - Description and significance of the problem/phenomenon studied; review of relevant theory and empirical work; problem statement	1-2
**Purpose or research question** - Purpose of the study and specific objectives or questions	1-2

Methods

**Qualitative approach and research paradigm** - Qualitative approach (e.g., ethnography, grounded theory, case study, phenomenology, narrative research) and guiding theory if appropriate; identifying the research paradigm (e.g., postpositivist, constructivist/ interpretivist) is also recommended; rationale**	2
**Researcher characteristics and reflexivity** - Researchers' characteristics that may influence the research, including personal attributes, qualifications/experience, relationship with participants, assumptions, and/or presuppositions; potential or actual interaction between researchers' characteristics and the research questions, approach, methods, results, and/or transferability	/
**Context** - Setting/site and salient contextual factors; rationale**	2
**Sampling strategy** - How and why research participants, documents, or events were selected; criteria for deciding when no further sampling was necessary (e.g., sampling saturation); rationale**	2
**Ethical issues pertaining to human subjects** - Documentation of approval by an appropriate ethics review board and participant consent, or explanation for lack thereof; other confidentiality and data security issues	2
**Data collection methods** - Types of data collected; details of data collection procedures including (as appropriate) start and stop dates of data collection and analysis, iterative process, triangulation of sources/methods, and modification of procedures in response to evolving study findings; rationale**	2

For peer review only

For peer review only - http://bmjopen.bmj.com/site/about/guidelines.xhtml

**Data collection instruments and technologies** - Description of instruments (e.g., interview guides, questionnaires) and devices (e.g., audio recorders) used for data collection; if/how the instrument(s) changed over the course of the study	2
**Units of study** - Number and relevant characteristics of participants, documents, or events included in the study; level of participation (could be reported in results)	2
**Data processing** - Methods for processing data prior to and during analysis, including transcription, data entry, data management and security, verification of data integrity, data coding, and anonymization/de-identification of excerpts	2
**Data analysis** - Process by which inferences, themes, etc., were identified and developed, including the researchers involved in data analysis; usually references a specific paradigm or approach; rationale**	2
**Techniques to enhance trustworthiness** - Techniques to enhance trustworthiness and credibility of data analysis (e.g., member checking, audit trail, triangulation); rationale**	/

Results/findings

**Synthesis and interpretation** - Main findings (e.g., interpretations, inferences, and themes); might include development of a theory or model, or integration with prior research or theory	2-4
**Links to empirical data** - Evidence (e.g., quotes, field notes, text excerpts, photographs) to substantiate analytic findings	2-4

Discussion

**Integration with prior work, implications, transferability, and contribution(s) to the field -** Short summary of main findings; explanation of how findings and conclusions connect to, support, elaborate on, or challenge conclusions of earlier scholarship; discussion of scope of application/generalizability; identification of unique contribution(s) to scholarship in a discipline or field	4-5
**Limitations** - Trustworthiness and limitations of findings	5

Other

**Conflicts of interest** - Potential sources of influence or perceived influence on study conduct and conclusions; how these were managed	6
**Funding** - Sources of funding and other support; role of funders in data collection, interpretation, and reporting	6

*The authors created the SRQR by searching the literature to identify guidelines, reporting standards, and critical appraisal criteria for qualitative research; reviewing the reference lists of retrieved sources; and contacting experts to gain feedback. The SRQR aims to improve the transparency of all aspects of qualitative research by providing clear standards for reporting qualitative research.

For peer review only - http://bmjopen.bmj.com/site/about/guidelines.xhtml

**The rationale should briefly discuss the justification for choosing that theory, approach, method, or technique rather than other options available, the assumptions and limitations implicit in those choices, and how those choices influence study conclusions and transferability. As appropriate, the rationale for several items might be discussed together.

Reference:

O'Brien BC, Harris IB, Beckman TJ, Reed DA, Cook DA. **Standards for reporting qualitative research: a synthesis of recommendations.** *Academic Medicine*, Vol. 89, No. 9 / Sept 2014 DOI: 10.1097/ACM.0000000000000388

For peer review only

For peer review only - http://bmjopen.bmj.com/site/about/guidelines.xhtml

BMJ Open

What are the Experiences of Teleophthalmology in Optometric Referral Pathways?: A Qualitative Interview Study with Patients and Clinicians.

Journal:	*BMJ Open*
Manuscript ID	bmjopen-2023-078161.R1
Article Type:	Original research
Date Submitted by the Author:	18-Dec-2023
Complete List of Authors:	Patel , Dilisha ; University College London, UCL Interaction Centre<br>Abdi, Sarah; University College London, UCL Interaction Centre<br>Carmichael, Josie; University College London, UCL Interaction Centre<br>Balaskas, Konstantinos; Moorfields Eye Hospital NHS Foundation Trust,<br>Blandford, Ann; University College London, UCL Interaction Centre
**Primary Subject Heading**:	Ophthalmology
Secondary Subject Heading:	Health services research, Qualitative research
Keywords:	Telemedicine < BIOTECHNOLOGY & BIOINFORMATICS, Public health < INFECTIOUS DISEASES, OPHTHALMOLOGY

SCHOLARONE™
Manuscripts

I, the Submitting Author has the right to grant and does grant on behalf of all authors of the Work (as defined in the below author licence), an exclusive licence and/or a non-exclusive licence for contributions from authors who are: i) UK Crown employees; ii) where BMJ has agreed a CC-BY licence shall apply, and/or iii) in accordance with the terms applicable for US Federal Government officers or employees acting as part of their official duties; on a worldwide, perpetual, irrevocable, royalty-free basis to BMJ Publishing Group Ltd ("BMJ") its licensees and where the relevant Journal is co-owned by BMJ to the co-owners of the Journal, to publish the Work in this journal and any other BMJ products and to exploit all rights, as set out in our licence.

The Submitting Author accepts and understands that any supply made under these terms is made by BMJ to the Submitting Author unless you are acting as an employee on behalf of your employer or a postgraduate student of an affiliated institution which is paying any applicable article publishing charge ("APC") for Open Access articles. Where the Submitting Author wishes to make the Work available on an Open Access basis (and intends to pay the relevant APC), the terms of reuse of such Open Access shall be governed by a Creative Commons licence – details of these licences and which Creative Commons licence will apply to this Work are set out in our licence referred to above.

Other than as permitted in any relevant BMJ Author's Self Archiving Policies, I confirm this Work has not been accepted for publication elsewhere, is not being considered for publication elsewhere and does not duplicate material already published. I confirm all authors consent to publication of this Work and authorise the granting of this licence.

For peer review only

What are the Experiences of Teleophthalmology in Optometric Referral Pathways?: A Qualitative Interview Study with Patients and Clinicians.

Dilisha Patel*, Sarah Abdi, Josie Carmichael, Konstantinos Balaskas and Ann Blandford.

*Corresponding author: Dr Dilisha Patel, UCL Interaction Centre, 66-72 Gower Street, London, WC1E 6EA, UK. Dilisha.patel@ucl.ac.uk,

Sarah Abdi, UCL Interaction Centre, 66-72 Gower Street, London, WC1E 6EA, UK. Sarah.abdi@ucl.ac.uk

Josie Carmichael, UCL Interaction Centre, 66-72 Gower Street, London, WC1E 6EA, UK. Josie.carmichael.20@ucl.ac.uk

Konstantinos Balaskas, Moorfields Eye Hospital NHS Foundation Trust, 162 City Road London, UK. EC1V 9PD. K.balaskas@nhs.net

Ann Blandford, UCL Interaction Centre, 66-72 Gower Street, London, WC1E 6EA, UK. A.blandford@ucl.ac.uk

Keywords: Teleophthalmology, Ophthalmology, Telemedicine, Referrals, Public Health,

Word Count: 2985

For peer review only

What are the Experiences of Teleophthalmology in Optometric Referral Pathways?: A Qualitative Interview Study with Patients and Clinicians.

Abstract

OBJECTIVE: Implementing teleophthalmology into the optometric referral pathway may ease the current pressures on hospital eye services caused by over-referrals from some optometrists. This study aimed to understand the practical implications of implementing teleophthalmology by analysing lived experiences and perceptions of teleophthalmology in the optometric referral pathway for suspected retinal conditions.

DESIGN: Qualitative In-depth Interview Study

SETTING: Fourteen primary care optometry practices and four secondary care hospital eye services.

PARTICIPANTS: We interviewed 41 participants: patients (17), optometrists (18), and ophthalmologists (6) who were involved in the HERMES Study. Through thematic analysis, we collated and present their experiences of implementing teleophthalmology.

RESULTS: All participants interviewed were positive towards teleophthalmology as it could enable efficiencies in the referral pathway and improve feedback and communication between patients and healthcare professionals. Concerns included setup costs for optometrists and anxieties from patients about not seeing an ophthalmologist face-to-face. However, reducing unnecessary visits and increasing the availability of resources and capacity were seen as significant benefits.

CONCLUSIONS: Overall, we report positive experiences of implementing teleophthalmology into the optometric referral pathway for suspected retinal conditions. Successful implementation will require appropriate investment to set up and integrate new technology and remunerate services, and continued evaluation to ensure timely feedback to patients and between healthcare professionals is received.

Strengths and Limitations:

- The strengths of our study include our ability to report on participants' rich and compelling lived experiences through in-depth qualitative interviews of using and being involved in referral pathways that used teleophthalmology over prospective opinions about the technology.
- We can further qualify previous literature that has reported teleophthalmology's potential impacts and benefits with these lived experiences within the HERMES study.
- This study collected data from multiple stakeholders, including primary and secondary eye care professionals and patients.
- A limitation of this study was that all ophthalmologists interviewed were involved in the HERMES study and, therefore may have had more knowledge of teleophthalmology than others.

Introduction

Primary eyecare in the U.K. is mainly delivered by community optometry practices [1], [2]. If patients are suspected of having a retinal condition, referrals to hospital eye services (HES) are typically processed by their general practitioner (G.P.) based on recommendations from the community-based optometrist. Thus, optometrists are often not involved in making direct referrals using electronic referral platforms or informed of outcomes. The additional step can reduce the specificity of clinical details included in the referral, as GPs are not specialists in eye care and rarely have the time or expertise to undertake eye examinations [3]. For several reasons, including concerns over capacity, changes in practice due to the Covid-19 pandemic, and the high number of referrals to HES, there is a need

For peer review only - http://bmjopen.bmj.com/site/about/guidelines.xhtml

for disruptive changes in the optometric referral pathways for suspected retinal conditions (SRC)[4]. We explore the experiences of patients, optometrists, and ophthalmologists of teleophthalmology for SRC.

Teleophthalmology describes the process of providing health information with medical technology at a distance, geographical, temporal or both [5], [6], to facilitate decision-making. In our study, we focused on an asynchronous platform; this allowed through uploading clinical information and multi-modal retinal imaging, i.e., fundus photography and macular OCT scans directly from the optometry practice, for later review by the receiving HES-based ophthalmologists. A benefit of the custom-built study platform, when compared to recent attempts for the commissioning of teleophthalmology-like store-and-forward referral pathways, is that it enables optometrists to upload full-volume optical coherence tomography (OCT) scans from multiple device manufacturers, in both their proprietary and open-source file format, which can then be parsed and reconstructed into a high-quality full volume scan, directly viewable on the platform's embedded viewer [7], [8]. Given the critical role of OCT imaging for diagnosing and managing medical retina conditions, a minimum clinical dataset including full-volume OCT is a prerequisite for the safe and efficient delivery of teleophthalmology referral triaging, providing ophthalmologists with essential information to confidently make referral triaging decisions. In the absence of teleophthalmology, or when attempts to implement teleophthalmology-like pathways are made with minimal input from clinical informatics and HES-based clinician and imaging experts, triaging ophthalmologists receive referrals, either without any accompanying imaging or frequently with one or a few selected cross-sectional images, not the entire volume [9], then referred patients are seen face to face to assess whether further investigation or treatment is needed.

The non-urgent referral pathway with and without teleophthalmology is presented in Figure 1. A recent systematic review reported that teleophthalmology and digital referrals could reduce waiting time, costs, and unnecessary referrals. It also noted that teleophthalmology could lead to earlier detection and diagnosis [10] and as such is an underutilised resource for HES. This review was based on reviewing referrals, not people's first-hand experiences. Therefore, there is a need to understand how implementation would affect users in practice.

Reports have shown that the growing use of OCT scanning has increased the number of referrals to HES [11], [12]; therefore, teleophthalmology has been suggested to reduce unnecessary referrals, manage growing capacity concerns and potentially manage increasing workloads by reviewing referrals before patients are seen in clinics.

We present findings from a study linked with a cluster randomised clinical trial (HERMES) evaluating the effectiveness of a teleophthalmology platform [1]. Those in the intervention arm of the trial were using teleophthalmology to refer patients to HES, while those in the control arm were using their regular referral pathways. We report the novel qualitative findings of patients' and healthcare professionals' experiences to understand the practical implications of implementing teleophthalmology.

Materials and Method

This study's overall aims, objectives and recruitment strategy are detailed elsewhere [13] ; we summarise key points here. We undertook semi-structured interviews with 41 participants between December 2021 and May 2022. These comprised 6 ophthalmologists who were all participating in the study and making remote referral decisions; 18 community optometrists, of whom 12 were from the intervention arm and 6 from the control arm of the HERMES study; and 17 patients recruited from community practices, of which 14 were from clinics in the intervention arm and 3 from clinics in the control arm. A semi-structured topic guide was used for all interviews and was tailored to each participant group, (included as supplementary material). All interviews were audio-recorded with consent. Five interviews were manually transcribed verbatim to aid familiarisation; the remaining interviews were transcribed using Scrintal Software. All transcripts were anonymised and checked for accuracy. The transcripts were coded using NVivo by an independent researcher who did not conduct the interviews, using inductive thematic analysis methods [14], [15]. 35 codes were initially defined and discussed with the research team. These were then refined and categorised into overarching themes. We present the three main themes which explore experiences of teleophthalmology for referrals for SRC.

For peer review only

Patients and Public Involvement

Eighteen patients were consulted in the planning and development of the wider HERMES Study, which has been detailed in the protocol paper [13]. The insights raised about understanding the benefits of teleophthalmology contributed to the design of this qualitative study. As noted above, 17 patients participated in interviews.

Results

We present our findings under three themes, *Efficiencies of Teleophthalmology; Teleophthalmology enables Feedback* and *Concerns about Teleophthalmology.* We found most participants were optimistic about the implementation of teleophthalmology in the optometric referral pathway due to the efficiencies the platform would enable. All participants expressed needing feedback during the referral process to improve care and highlighted some concerns.

Efficiencies of Teleophthalmology

All welcomed Teleophthalmology due to its ability to improve patient and clinician experiences. There is regional variation in referral pathways depending on the specific condition; GPs typically process routine referrals for SRC. However, it was reported that GPs were not always suited to create referrals due to their limited skillset in specialist eyecare. Patients often described GPs as the unnecessary "middleman". Patients reported being keen to be referred directly by their optometrist and felt this would reduce their waiting time to hear back from HES if referrals were processed directly.

"If the optician can do the referral directly rather than you know, the optician telling me you'll need to go and see your GP who will refer you (…) I would be more comfortable because they [optometrist] know what they're doing whereas the GP is just saying you are alright then, if your optician told you that, then I'll send you." (Patient 11)

In the HERMES study, optometrists used teleophthalmology to refer patients to HES, enabling a quicker referral process direct to triaging ophthalmologists. They shared that teleophthalmology could improve patient satisfaction and help relieve hospital capacity pressures by reducing unnecessary hospital visits.

"I think the whole teleophthalmology thing will improve patient satisfaction and it makes life a lot easier, less patients, elderly patients having to find transport to the hospital, and being dilated once in the optometry practice and then being dilated again, back at the hospital and patient transport having to be arranged, so overall good, big saving of cost and finance and less crowded waiting rooms at the hospital." (Optometrist 18)

Ophthalmologists shared that the teleophthalmology platform introduced uniformity to the referral process by requiring the same data fields to be completed for each patient, which was easy for the referring optometrist. This enabled referrals to be triaged and reviewed more quickly: referral decisions could be made promptly, and the appropriate triaging decision could be made regarding the indication and the urgency for a hospital visit.

"It's more informative because, as you know, the platform has the questions with the tick box, um, on the optician findings which [is] not always involved in a classic referral proforma. And obviously, it has the imaging as well, which helps us to make a decision very quickly." (Ophthalmologist 1)

As mentioned, the key benefit of the teleophthalmology platform is the ability to review and triage patient referrals without them having to attend a face-to-face appointment. All participants recognised and shared the advantage of saving time and resources by using teleophthalmology.

Teleophthalmology Enables Feedback

Some patients reported that they were happy for teleophthalmology to be used to assess their referrals as they would not want to attend HES if not required. Still, they expected feedback to explain the reason for not being seen for a

For peer review only

face-to-face appointment. This was not always provided. Some patients reported dissatisfaction with their referral experience due to the lack of communication. This was also shared in the context of not receiving feedback promptly. Patients expected to hear about their referral decision more promptly through the teleophthalmology process, which increased concerns over their eye health when this expectation was not satisfied. In these cases, patients wanted to be seen or told directly and promptly why an appointment was not required and not to be kept waiting in uncertainty. Teleophthalmology can overcome this expectation discrepancy through accurate information presentation and timely and clear feedback.

"If the patients were to get a letter or some form of communication from the hospital or the specialist to reassure them that your case has been looked at and this is what has been concluded, I think that would be enough to put somebody's mind at ease." (Patient 10)

Optometrists stated that one of the significant benefits of teleophthalmology was the ability to receive feedback from ophthalmologists. Optometrists reported that when patients were referred to HES in the absence of teleophthalmology, patients would often return to them seeking more information and advice about their care; therefore, it was important for optometrists to be involved in the referral pathway and remain informed of their patients' management plans. Optometrists shared that many patients were not a reliable source of information about their eye health/treatment, which could affect future care or monitoring they provided.

"Once we refer the patient, we don't actually know then what is happening thereafter, unless we chase the patient or, our patients are quite loyal, so they would, we would see them a year later, we will say or remember, we referred you last time, what happened? (...) So, it's actually we're basing it of what the patient is then telling us, so we are actually getting like the second story through the patient rather than the actual clinical information." (Optometrist 12)

By receiving feedback, optometrists can also verify whether their referrals were appropriate and audit themselves to improve the quality of their referrals.

"Because if we keep referring something that we think is urgent, but [the] ophthalmologist tells us this is not urgent, and if you learn by that, that's going to help you, you see. Right now, there's no feedback. (...). But if I got feedback from the ophthalmologist that saw the patient and I will know for next time when I see that similar sort of situation that well, actually this isn't urgent." (Optometrist 10)

Having a system where the community-based clinic is connected to the HES was also seen as a great benefit for ophthalmologists. They welcomed being able to provide feedback to the referring optometrists, especially to enable the sharing of referral decisions directly and concurred with the need to provide feedback on the referral quality to improve future referrals. It was suggested that the teleophthalmology system should send referral replies to the referring optometrist, patient, and GP so all are informed of the outcome.

Concerns about Teleophthalmology

There were some concerns about using technology to manage patient referrals. Some patients still wanted the reassurance of seeing a clinician rather than having their referral decision and notification completed remotely. Seeing someone face-to-face provided the holistic care some patients reported wanting and addressed their worries and anxieties.

“I think [if] you don't get a chance to see the patient yourself, there is something about looking at data visually transactionally, that is fine, but there is also something about talking to the patients about how they're feeling and how they're coping with things.” (Patient 10)

Optometrists were mainly concerned with the practicalities of implementing a new system into their workflow. This included concerns over training to use the equipment, the reliability of network connectivity and equipment costs

For peer review only

that some smaller practices may not be able to bear, as well as remuneration for their time for taking on additional roles. Some also reported that completing a referral on the teleophthalmology platform took time.

"The barriers would be cost, because this is all based on the information that is being sent from an OCT device, yeah, as part of the process of referral, it's not just from a letter, so when it comes to having the equipment, that's an immediate barrier. And having the right remuneration for the equipment." (Optometrist 2)

Ophthalmologists also shared these concerns; however, they were positive towards the ability of teleophthalmology, enabling them to use their time more efficiently.

Discussion

Whilst previous work has focused on the efficacy and efficiency of teleophthalmology platforms through reviewing referrals [12], [16], we report insights based on experiences from patients, optometrists, and ophthalmologists to validate previous findings on perceptions of using teleophthalmology for suspected retinal conditions.

All participants recognised the value of implementing a teleophthalmology system into their ophthalmic care pathway due to its potential to improve patient care and health services efficiencies. This is supported by others [17], who found through a review of referrals that teleophthalmology can reduce unnecessary HES visits and significantly impact patient anxieties [18]. Our study has shown this in practice, with many patients sharing that they would not want to attend HES if not required.

Both optometrists and ophthalmologists reported that teleophthalmology's significant advantage is the ability to electronically refer patients directly from optometrist practices to hospital eye services, which can significantly reduce the waiting time for patients. The ability to triage referrals electronically also enabled ophthalmologists to provide replies and feedback to the referring optometrist via the teleophthalmology platform, which they greatly valued.

Implementing teleophthalmology into the eye care pathway would remove the burden from GPs of having to process patient referrals, but they must be informed of such referrals. GPs have, in principle, supported the suggestion of optometrists referring patients directly [16], [19], and we found that patients and optometrists would support this change in practice. There is great value in involving optometrists in the referral process, as it has been reported that this could reduce unnecessary referrals by approximately half [12]

The overarching theme shared by all participants that substantiates many of the benefits of the teleophthalmology platform is the potential of the platform to facilitate the provision of feedback. The importance of receiving timely feedback in eye care, in general, has been reported by others [3], [20] and was essential for patients to alleviate their concerns over their eye health. Harvey et al. (2022) specifically outlines the factors that could affect the provision of feedback, and a key implication of their work is the call for technology to support this provision. Thus, we found that teleophthalmology could overcome concerns in the optometric referral pathway.

Whilst feedback can keep both patients and optometrists informed [21], it can also improve future referral quality through open conversations between referring clinicians. Our results concur as we report optometrists greatly value receiving referral replies directly from ophthalmologists to remain informed of their patients' care and audit their referrals. Additionally, previous research has highlighted that the lack of communication between optometrists and ophthalmologists can be problematic [22], [23]; therefore, implementing a teleophthalmology platform could help to overcome this.

Potential barriers raised by some optometrists were the initial setup costs, which include time, training, and financial costs. Whilst others have reported this should be considered [10], [16], we found this to be a key concern in practice. According to the current General Ophthalmic Services contract (2023), OCT scans are not a contracted service in optometric care [24]. Therefore, there is a need to ensure optometrists are appropriately remunerated for providing this service. Optometrists would also need to have appropriate access to NHS e-referral systems to expediently refer patients to the HES system. Others have begun to explore the cost-effectiveness of implementing teleophthalmology

For peer review only

systems [25]; further work is needed to establish health-economic benefits concerning SRC that were raised in our study.

To successfully implement teleophthalmology into the optometric referral pathway, there needs to be an investment to enable parity amongst optometry practices to support new technology setup. Whilst the implementation of teleophthalmology was perceived to initially increase the workload of optometrists to process referrals and ophthalmologists to triage referrals, with the correct remuneration, there is significant potential to relieve pressures on stretched eye care services.

We acknowledge study limitations including the influence of participation bias. The participants who chose to participate in our research may have different views from those who did not participate. However, through in-depth interviews, we were able to elicit the lived experiences of those involved in the study, some of whom had direct experience with the teleophthalmology platform.

Conclusions

Implementing teleophthalmology into the optometric referral pathway has numerous benefits, as outlined by all participants. Through our in-depth interview study with patients, optometrists, and ophthalmologists, we found that they generally report great value in implementing teleophthalmology through improving efficiency and the ability to provide and receive feedback. Patients were satisfied if their referrals were reviewed with teleophthalmology to reduce the possibility of unnecessary HES visits if this was clearly and efficiently communicated back to them. Optometrists felt they were better suited than GPs to write and process patient referrals and would feel more valued if they were more directly involved in the pathway. Finally, ophthalmologists were pleased with a system enabling them to manage their caseloads more efficiently. Further efficiencies teleophthalmology can promote include removing the burden on GPs, the time patients wait to be seen by HES, the time it takes for ophthalmologists to review and provide referral replies and finally, the overarching benefit to all participants of being involved and receiving feedback. Future work could explore how to overcome barriers such as connectivity and the specific health economics of implementing teleophthalmology to validate our findings.

Acknowledgements

We thank all those involved in this research, including participants and the wider research team.

Funding Statement

This work was funded by NIHR Health Technology Assessment grant number 18/182.

Competing Interests

We have no competing interests to declare.

Author Contributions

DP analysed the data and prepared the manuscript. SA, KB & AB designed the study protocol, SA undertook recruitment and data collection. SA, JC & AB contributed analysis as presented by DP. All authors reviewed and contributed to subsequent drafts and approved the final manuscript.

Ethics Statement

Ethical approvals were gained from London-Bromley Research Ethics Committee (REC 20/LO/1299). Informed consent was obtained from participants after details of the study, including the aims and methods, were disclosed.

For peer review only

Data Availability Statement

We are happy to be contacted to share anonymised data. Please contact the corresponding or senior author for requests.

References

[1] J. E. D. Han and others, 'Teleophthalmology-enabled and artificial intelligence-ready referral pathway for community optometry referrals of retinal disease (HERMES): a Cluster Randomised Superiority Trial with a linked Diagnostic Accuracy Study-HERMES study report 1-study protocol', *BMJ Open*, vol. 12, no. 2, p. e055845, 2022.

[2] B. Bowling, S. D. M. Chen, and J. F. Salmon, 'Outcomes of referrals by community optometrists to a hospital glaucoma service', *Br J Ophthalmol*, vol. 89, no. 9, pp. 1102–1104, Sep. 2005, doi: 10.1136/bjo.2004.064378.

[3] T. Pierscionek, J. Moore, and B. Pierscionek, 'Referrals to ophthalmology: optometric and general practice comparison', *Ophthalmic and Physiological Optics*, vol. 29, no. 1, pp. 32–40, 2009.

[4] Y. C. Tham and others, 'New digital models of care in ophthalmology, during and beyond the COVID-19 pandemic', *Br J Ophthalmol*, vol. 106, no. 4, pp. 452–457, 2022.

[5] L. J. Caffery and others, 'Models of care in tele-ophthalmology: A scoping review', *J Telemed Telecare*, vol. 25, no. 2, pp. 106–122, 2019.

[6] T. S. Surendran and R. Raman, 'Teleophthalmology in Diabetic Retinopathy', *J Diabetes Sci Technol*, vol. 8, no. 2, pp. 262–266, 2014.

[7] 'Improve referrals to eye care services', NHS Transformation Directorate. Accessed: Dec. 09, 2023. [Online]. Available: https://transform.england.nhs.uk/key-tools-and-info/digital-playbooks/eye-care-digital-playbook/improve-referrals-to-eye-care-services/

[8] C. Roberts, 'How digital eye care referrals will transform care'. 2021.

[9] H. a. S. C. Committee, 'Evaluation of Government commitments made on the digitisation of the NHS. Fourth Special Report of Session 2022-23', 2023.

[10] M. P. Conway and others, 'Investigating the Role of Optometrists in Teleophthalmology and the Implications of Increasing Access to Advanced Imaging Techniques and Digital Referral: A Systematic Search and Review', *Telemedicine and e-Health*, vol. 27, no. 9, pp. 974–981, 2021.

[11] J. X. Lee, V. Manjunath, and S. J. Talks, 'Expanding the role of medical retina virtual clinics using multimodal ultra-widefield and optical coherence tomography imaging', *Clin Ophthalmol*, vol. 12, pp. 2337–2345, 2018.

[12] C. Kern and others, 'Implementation of a cloud-based referral platform in ophthalmology: making telemedicine services a reality in eye care', *Br J Ophthalmol*, vol. 104, no. 3, pp. 312–317, 2020.

[13] A. Blandford and others, 'Protocol for a qualitative study to explore acceptability, barriers and facilitators of the implementation of new teleophthalmology technologies between community optometry practices and hospital eye services', *BMJ Open*, vol. 12, no. 7, p. e060810, 2022.

[14] V. Braun and V. Clarke, 'Using Thematic Analysis in Psychology', *Vol. 3*, pp. 77–101, 2006.

[15] V. Braun and V. Clarke, *Successful qualitative research: A practical guide for beginners*. Sage, 2013.

[16] S. P. Kelly and others, 'Teleophthalmology with optical coherence tomography imaging in community optometry. Evaluation of a quality improvement for macular patients', *Clinical Ophthalmology*, vol. 5, p. 1673, 2011.

[17] D. V. Muttuvelu and others, 'Danish teleophthalmology platform reduces optometry referrals into the national eye care system', *BMJ Open Ophthalmology*, vol. 6, no. 1, p. e000671, 2021.

[18] C. J. Davey, C. Harley, and D. B. Elliott, 'Levels of state and trait anxiety in patients referred to ophthalmology by primary care clinicians: a cross-sectional study', *PLoS One*, vol. 8, no. 6, p. e65708, 2013.

[19] NHS, '2023/24 priorities and operational planning guidance', Jan. 2023. Accessed: Dec. 09, 2023. [Online]. Available: https://www.england.nhs.uk/wp-content/uploads/2022/12/PRN00021-23-24-priorities-and-operational-planning-guidance-v1.1.pdf

[20] K. Harvey and others, 'Referrals from community optometrists in England and their replies: A mixed methods study', *Ophthalmic and Physiological Optics*, vol. 42, no. 3, pp. 454–470, 2022.

[21] D. M. Baldwin and others, 'Patient preferences for notification of normal laboratory test results: a report from the ASIPS Collaborative', *BMC family practice*, vol. 6, no. 1, pp. 1–7, 2005.

[22] A. S. O�Malley and J. D. Reschovsky, 'Referral and consultation communication between primary care and specialist physicians: finding common ground', *Archives of internal medicine*, vol. 171, no. 1, pp. 56–65, 2011.

[23] D. Townsend and others, 'Health professionals� and service users� perspectives of shared care for monitoring wet age-related macular degeneration: a qualitative study alongside the ECHoES trial', *BMJ open*, vol. 5, no. 4, p. e007400, 2015.
[24] N. England, 'General Ophthalmic Mandatory Services Model Contract', NHS, Sep. 2023. Accessed: Dec. 09, 2023. [Online]. Available: https://www.england.nhs.uk/wp-content/uploads/2018/08/PRN00242-General-ophthalmic-mandatory-services-model-contract-September-2023.pdf
[25] N. Sharafeldin and others, 'Review of economic evaluations of teleophthalmology as a screening strategy for chronic eye disease in adults', *Br J Ophthalmol*, vol. 102, no. 11, pp. 1485–1491, 2018.

List of Figures

Figure 1: The non-urgent patient referral pathway with and without

For peer review only

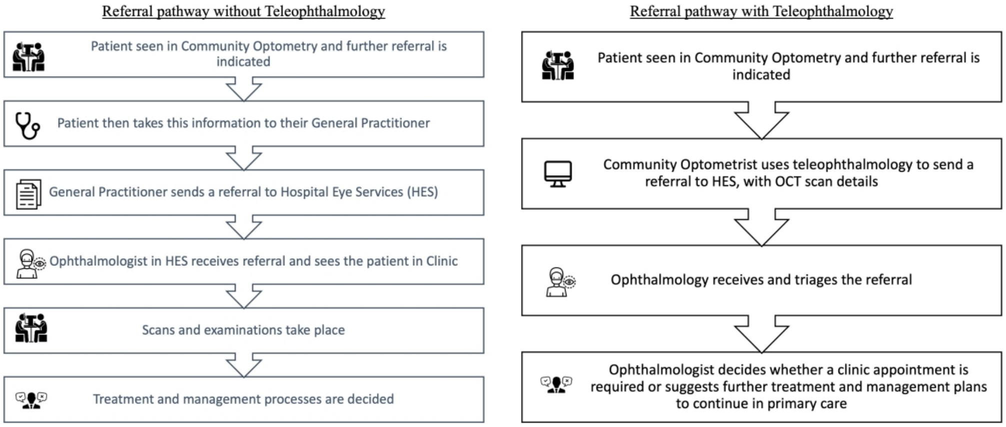

317x135mm (330 x 330 DPI)

Interview topic guide- Patients

1. Information and consent

- Briefly re-introduce the topic, the purpose of the overall project and the specific aim of the HCI study
- Provide assurances regarding confidentiality and how information will be processed
- Check willingness to continue to participate
- Obtain informed consent (if not taken in advance) and permission to be audio/video recorded

2. Introduction

Thank you for taking part in this interview. We really appreciate you taking the time to talk with us. As I explained earlier, we are keen to talk to you about the use of new tele-eye care technologies in the referral process between community optometric practices and hospital eye services. We want to hear your views on these technologies and whether they would work for you in either a good or bad way. There are no right or wrong answers, so feel free to tell us what you think, and if there is something that needs to be improved.

The interview is likely to take around 30 minutes, you are free to stop and take a break whenever you feel like. It is also voluntary, which means you can opt out at any time for any reason.

3. Background information

- So, first of all: I would like to know more about you and your use of primary and secondary eye services.
- How old are you?
- Do you have any known eye problems?
- How often do you visit your optician?
  - Do you do regular check-ups, or do you visit the practice when you have eye problems only?
  - When was your last visit to optometric practice [i.e., the one before your most recent appointment)?

For peer review only

- What led to you to making your most recent eye appointment [i.e., the one that has led to the referral]?
- What did you expect to happen during that particular appointment?
- Have you been referred to hospital for eye care before?

4. Questions related to the teleophthalmology platform

- Can you describe to me your most recent visit to the optometric practice services? [i.e., the one you just had with the OCT scan?]
  - Can you describe to me the steps that you went through during the consultation with the optometrist?
  - **For the intervention arm:**
    - Have you noticed any difference between the care you received this time and last time? Were you asked to do something different during this consultation to what you were used to do? What was it? What are your thoughts on this?
  - Were the OCT scan results discussed with you? When were you informed about the referral decision?
  - What were you told will happen next? How did you feel about that?
  - How long did you tell you it will take to see a doctor?
- **For the intervention arm**
- Have you **received feedback** about your referral?
  - How long did it take from the time you were referred by your optician to the time you received the feedback (referral letter)? What do you think about this waiting time?
  - What were the information included in the feedback letter? (do you remember?), what do you think about that?
  - What happened next?
- **For the control arm**
  - What do you think a tele-eye care service or a teleophthalmology service in the referral process is? **Explain what teleophthalmology is in the context of the HERMES project.**
- Generally, what do you think about this new referral process?
- What do you think the benefits of this service are?
- What do you think the problems with this service are?

- What do you think the disadvantages would be for you as a patient if this type of service was implemented in real life?
- In this type of service, an optician at the community optometry practice and an ophthalmologist at the hospital will be involved in the referral process, who would you be most confident in their decision? And why?
- How would you feel if you were referred to hospital for eye care when it was not necessary?
    - How would you feel spending this time on a visit that wasn't necessary?
    - What do you think should be done to avoid referring you when it is not necessary?
- Some of the opticians refer all cases with suspected retinal conditions to hospital, which may include those who are urgent and those that turn out to be unnecessary (unnecessary referrals). So when hospital receive these referrals, the hospital has two options- **Option 1:** See all patients referred by opticians and provide re-assurances to everyone regarding their eye conditions or **Option 2:** hospital sees mainly urgent cases who require hospital care.
    - What do you think of these options? Which one would you prefer? Why?

6. End of the interview

We have reached the end of the interview. Would you like to share any more ideas or thoughts?

Thank you so much for taking the time to participate in the study, we will use this information to inform the design of a future tele-eye care service.

Interview topic guide – Primary care professionals - Control

1. Information and consent

- Briefly re-introduce the topic, the purpose of the overall project and the specific aim of the HCI study
- Obtain informed consent (if not taken in advance) and permission to be audio/video recorded

For peer review only

2. Introduction

Thank you for taking part in this interview. We really appreciate you taking the time to talk with us. As I explained earlier, we are keen to talk to you about the use of new teleophthalmology technologies in the referral process between community optometry practices and hospital eye services. We want to hear your views on these technologies, and what factors you think may influence their implementation in real life**.** There are no right or wrong answers, so feel free to tell us what you think, and if there is something that needs to be improved.

The interview is likely to take around 30-45 minutes. Your participation is voluntary, and you can withdraw from the study at any time.

3. Background Information

- First of all: I will start by asking you a few questions to know more about you.
- What is your professional background?
- How long have you been working in community optometry practices?
- How often do you refer patients with retinal conditions to hospital eye services? (in a week what is the average number of cases identified with retinal conditions)
- Can you explain to me the different referral pathways that you follow when you identify someone with a suspected retinal condition (usual not through the teleophthalmology platform)
- What do you think of the referral process?
    - What are the issues or barriers that you face when referring patients?
- How do you think the referral process can be improved?

4. Teleophthalmology platform

4.1 Expectation/coherence

- What do you think a teleophthalmology platform is? How different is it from the traditional system?
- What would you like to see in a teleophthalmology platform?
- How do you think a teleophthalmology platform would benefit/improve the referral process for eye conditions?

For peer review only

- What barriers do you think there will be to using a teleophthalmology platform in the context of referrals?
- What do you think would need to change to incorporate a teleophthalmology platform in your practice?

6. End of the interview

We have reached the end of the interview. Would you like to share any more ideas or thoughts?

Thank you so much for taking the time to participate in the study, we will use this information to inform the design of future teleophthalmology services.

Interview topic guide – Primary care professionals - Intervention

1. Information and consent

- Briefly re-introduce the topic, the purpose of the overall project and the specific aim of the HCI study
- Obtain informed consent (if not taken in advance) and permission to be audio/video recorded

2. Introduction

Thank you for taking part in this interview. We really appreciate you taking the time to talk with us. As I explained earlier, we are keen to talk to you about the use of new teleophthalmology technologies in the referral process between community optometry practices and hospital eye services. We want to hear your views on these technologies, and what factors you think may influence their implementation in real life**.** There are no right or wrong answers, so feel free to tell us what you think, and if there is something that needs to be improved.

For peer review only

The interview is likely to take around 30-45 minutes. Your participation is voluntary, and you can withdraw from the study at any time.

3. Background Information

- First of all: I will start by asking you a few questions to know more about you.
- What is your professional background?
- How long have you been working in community optometry practices?
- How often do you refer patients with retinal conditions to hospital eye services? (in a week what is the average number of cases identified with retinal conditions)
- Can you explain to me the different referral pathways that you follow when you identify someone with a suspected retinal condition (usual not through the teleophthalmology platform)
- What do you think of the referral process?
    - What are the issues or barriers that you face when referring patients?
- How do you think the referral process can be improved?

4. Teleophthalmology platform

4.1 Experience/cognitive participation

- How long have you been using this new teleophthalmology platform?
- Can you describe to me the steps you follow when you refer a patient with a suspected retinal condition through the new teleophthalmology platform?
- How long does it take you to do a referral on this platform? What do you think of that?
- What did you like about this new platform? Why?
- What did you not like about this new platform? Why?
    - What were the issues that you faced when using the platform?
    - Do you have any concerns regarding the use of this platform?
- What type of support did you receive to use this platform (e.g., training, support staff)?

4.2 Experience/collective action

- What changes did you have to make in your work routine to incorporate this new technology?
- How will this new platform fit in your current practice?
- How do you think this new platform will impact/improve your practice, patient care and your working relationship with secondary care?

For peer review only

4.3 Reflective monitoring

- What do you think will be the benefits of this new teleophthalmology platform on the referral process, both short term and long term?
    - How do you think this new teleophthalmology platform can help reduce unnecessary referrals?
    - How do you think this new teleophthalmology platform can help improve the quality of referrals?
- What barriers/disadvantages do you think there will be to using this new platform on the referral process?
- What do you think would need to change for this technology to be successfully embedded in routine eye practice (e.g., training…)?

6. End of the interview

We have reached the end of the interview. Would you like to share any more ideas or thoughts?

Thank you so much for taking the time to participate in the study, we will use this information to inform the design of future teleophthalmology services.

<u>Questions for ophthalmologists:</u>

- I will be asking you a few questions regarding the use of the new teleophthalmology platform in the referral process.
- I will audio record the session, are you happy for me to do so? Consent form points.
    1. Can you sketch out the overall referral process for retinal conditions and how it works from your trust's perspective?
    2. What are the main issues you face in the referral process from community optometry practices?
    3. About how many new retinal referrals do you deal with per week? Usually how do you organize this aspect of your work? ** Usual practice**
    4. For the HERMES platform, how are you notified about new referrals on the system? How often do you check the system for new referrals, or how soon do you review the referral? How many referrals do you receive in this system per week?
    5. How long does it take you to review each referral? What do you think of this duration? Reasonable, good, long, can be improved?
    6. Who else check the referrals on the teleophthalmology platform at your trust? How do you coordinate your work with them? Do you distribute the referrals amongst

For peer review only

yourselves, or do you review each new referral? How do you validate your referral decision?

7. So far, what do you think about the quality of the referrals received by this platform? Have you observed any changes compared to usual route?
8. What are the things that you liked about the platform, things that are working well so far?
9. What are the pain points of the platform? What are the things that you did not like? And how can they be improved?
10. What happens next, after you decided on the referral?
11. Looking to the future, how do you envision that this system will be rolled out across your trust or are a couple of consultants always going to be able to assess all OCT scans?
12. What do you think would need to change for this technology to be successfully embedded in routine eye practice?

For peer review only

Standards for Reporting Qualitative Research (SRQR)*

http://www.equator-network.org/reporting-guidelines/srqr/

Title and abstract

	Page/line no(s).
**Title** - Concise description of the nature and topic of the study Identifying the study as qualitative or indicating the approach (e.g., ethnography, grounded theory) or data collection methods (e.g., interview, focus group) is recommended	1
**Abstract** - Summary of key elements of the study using the abstract format of the intended publication; typically includes background, purpose, methods, results, and conclusions	1

Introduction

**Problem formulation** - Description and significance of the problem/phenomenon studied; review of relevant theory and empirical work; problem statement	1-2
**Purpose or research question** - Purpose of the study and specific objectives or questions	1-2

Methods

**Qualitative approach and research paradigm** - Qualitative approach (e.g., ethnography, grounded theory, case study, phenomenology, narrative research) and guiding theory if appropriate; identifying the research paradigm (e.g., postpositivist, constructivist/ interpretivist) is also recommended; rationale**	2
**Researcher characteristics and reflexivity** - Researchers' characteristics that may influence the research, including personal attributes, qualifications/experience, relationship with participants, assumptions, and/or presuppositions; potential or actual interaction between researchers' characteristics and the research questions, approach, methods, results, and/or transferability	/
**Context** - Setting/site and salient contextual factors; rationale**	2
**Sampling strategy** - How and why research participants, documents, or events were selected; criteria for deciding when no further sampling was necessary (e.g., sampling saturation); rationale**	2
**Ethical issues pertaining to human subjects** - Documentation of approval by an appropriate ethics review board and participant consent, or explanation for lack thereof; other confidentiality and data security issues	2
**Data collection methods** - Types of data collected; details of data collection procedures including (as appropriate) start and stop dates of data collection and analysis, iterative process, triangulation of sources/methods, and modification of procedures in response to evolving study findings; rationale**	2

For peer review only

**Data collection instruments and technologies** - Description of instruments (e.g., interview guides, questionnaires) and devices (e.g., audio recorders) used for data collection; if/how the instrument(s) changed over the course of the study	2
**Units of study** - Number and relevant characteristics of participants, documents, or events included in the study; level of participation (could be reported in results)	2
**Data processing** - Methods for processing data prior to and during analysis, including transcription, data entry, data management and security, verification of data integrity, data coding, and anonymization/de-identification of excerpts	2
**Data analysis** - Process by which inferences, themes, etc., were identified and developed, including the researchers involved in data analysis; usually references a specific paradigm or approach; rationale**	2
**Techniques to enhance trustworthiness** - Techniques to enhance trustworthiness and credibility of data analysis (e.g., member checking, audit trail, triangulation); rationale**	/

Results/findings

**Synthesis and interpretation** - Main findings (e.g., interpretations, inferences, and themes); might include development of a theory or model, or integration with prior research or theory	2-4
**Links to empirical data** - Evidence (e.g., quotes, field notes, text excerpts, photographs) to substantiate analytic findings	2-4

Discussion

**Integration with prior work, implications, transferability, and contribution(s) to the field** - Short summary of main findings; explanation of how findings and conclusions connect to, support, elaborate on, or challenge conclusions of earlier scholarship; discussion of scope of application/generalizability; identification of unique contribution(s) to scholarship in a discipline or field	4-5
**Limitations** - Trustworthiness and limitations of findings	5

Other

**Conflicts of interest** - Potential sources of influence or perceived influence on study conduct and conclusions; how these were managed	6
**Funding** - Sources of funding and other support; role of funders in data collection, interpretation, and reporting	6

*The authors created the SRQR by searching the literature to identify guidelines, reporting standards, and critical appraisal criteria for qualitative research; reviewing the reference lists of retrieved sources; and contacting experts to gain feedback. The SRQR aims to improve the transparency of all aspects of qualitative research by providing clear standards for reporting qualitative research.

For peer review only - http://bmjopen.bmj.com/site/about/guidelines.xhtml

For peer review only

**The rationale should briefly discuss the justification for choosing that theory, approach, method, or technique rather than other options available, the assumptions and limitations implicit in those choices, and how those choices influence study conclusions and transferability. As appropriate, the rationale for several items might be discussed together.

Reference:

O'Brien BC, Harris IB, Beckman TJ, Reed DA, Cook DA. **Standards for reporting qualitative research: a synthesis of recommendations.** *Academic Medicine*, Vol. 89, No. 9 / Sept 2014 DOI: 10.1097/ACM.0000000000000388

For peer review only

For peer review only - http://bmjopen.bmj.com/site/about/guidelines.xhtml

BMJ Open

What are the Experiences of Teleophthalmology in Optometric Referral Pathways?: A Qualitative Interview Study with Patients and Clinicians.

Journal:	*BMJ Open*
Manuscript ID	bmjopen-2023-078161.R2
Article Type:	Original research
Date Submitted by the Author:	11-Jan-2024
Complete List of Authors:	Patel , Dilisha ; University College London, UCL Interaction Centre<br>Abdi, Sarah; University College London, UCL Interaction Centre<br>Carmichael, Josie; University College London, UCL Interaction Centre<br>Balaskas, Konstantinos; Moorfields Eye Hospital NHS Foundation Trust,<br>Blandford, Ann; University College London, UCL Interaction Centre
**Primary Subject Heading**:	Ophthalmology
Secondary Subject Heading:	Health services research, Qualitative research
Keywords:	Telemedicine < BIOTECHNOLOGY & BIOINFORMATICS, Public health < INFECTIOUS DISEASES, OPHTHALMOLOGY

SCHOLARONE™
Manuscripts

I, the Submitting Author has the right to grant and does grant on behalf of all authors of the Work (as defined in the below author licence), an exclusive licence and/or a non-exclusive licence for contributions from authors who are: i) UK Crown employees; ii) where BMJ has agreed a CC-BY licence shall apply, and/or iii) in accordance with the terms applicable for US Federal Government officers or employees acting as part of their official duties; on a worldwide, perpetual, irrevocable, royalty-free basis to BMJ Publishing Group Ltd ("BMJ") its licensees and where the relevant Journal is co-owned by BMJ to the co-owners of the Journal, to publish the Work in this journal and any other BMJ products and to exploit all rights, as set out in our licence.

The Submitting Author accepts and understands that any supply made under these terms is made by BMJ to the Submitting Author unless you are acting as an employee on behalf of your employer or a postgraduate student of an affiliated institution which is paying any applicable article publishing charge ("APC") for Open Access articles. Where the Submitting Author wishes to make the Work available on an Open Access basis (and intends to pay the relevant APC), the terms of reuse of such Open Access shall be governed by a Creative Commons licence – details of these licences and which Creative Commons licence will apply to this Work are set out in our licence referred to above.

Other than as permitted in any relevant BMJ Author's Self Archiving Policies, I confirm this Work has not been accepted for publication elsewhere, is not being considered for publication elsewhere and does not duplicate material already published. I confirm all authors consent to publication of this Work and authorise the granting of this licence.

For peer review only

What are the Experiences of Teleophthalmology in Optometric Referral Pathways?: A Qualitative Interview Study with Patients and Clinicians.

Dilisha Patel*, Sarah Abdi, Josie Carmichael, Konstantinos Balaskas and Ann Blandford.

*Corresponding author: Dr Dilisha Patel, UCL Interaction Centre, 66-72 Gower Street, London, WC1E 6EA, UK. Dilisha.patel@ucl.ac.uk,

Sarah Abdi, UCL Interaction Centre, 66-72 Gower Street, London, WC1E 6EA, UK. Sarah.abdi@ucl.ac.uk

Josie Carmichael, UCL Interaction Centre, 66-72 Gower Street, London, WC1E 6EA, UK. Josie.carmichael.20@ucl.ac.uk

Konstantinos Balaskas, Moorfields Eye Hospital NHS Foundation Trust, 162 City Road London, UK. EC1V 9PD. K.balaskas@nhs.net

Ann Blandford, UCL Interaction Centre, 66-72 Gower Street, London, WC1E 6EA, UK. A.blandford@ucl.ac.uk

Keywords: Teleophthalmology, Ophthalmology, Telemedicine, Referrals, Public Health,

Word Count: 3163

For peer review only

What are the Experiences of Teleophthalmology in Optometric Referral Pathways?: A Qualitative Interview Study with Patients and Clinicians.

Abstract

OBJECTIVE: Implementing teleophthalmology into the optometric referral pathway may ease the current pressures on hospital eye services caused by over-referrals from some optometrists. This study aimed to understand the practical implications of implementing teleophthalmology by analysing lived experiences and perceptions of teleophthalmology in the optometric referral pathway for suspected retinal conditions.

DESIGN: Qualitative In-depth Interview Study

SETTING: Fourteen primary care optometry practices and four secondary care hospital eye services from four NHS Foundation Trusts across the UK.

PARTICIPANTS: We interviewed 41 participants: patients (17), optometrists (18), and ophthalmologists (6) who were involved in the HERMES Study. Through thematic analysis, we collated and present their experiences of implementing teleophthalmology.

RESULTS: All participants interviewed were positive towards teleophthalmology as it could enable efficiencies in the referral pathway and improve feedback and communication between patients and healthcare professionals. Concerns included setup costs for optometrists and anxieties from patients about not seeing an ophthalmologist face-to-face. However, reducing unnecessary visits and increasing the availability of resources and capacity were seen as significant benefits.

CONCLUSIONS: Overall, we report positive experiences of implementing teleophthalmology into the optometric referral pathway for suspected retinal conditions. Successful implementation will require appropriate investment to set up and integrate new technology and remunerate services, and continued evaluation to ensure timely feedback to patients and between healthcare professionals is received.

Strengths and Limitations:

- The strengths of our study include our ability to report on participants' rich and compelling lived experiences through in-depth qualitative interviews of using and being involved in referral pathways that used teleophthalmology over prospective opinions about the technology.
- We can further qualify previous literature that has reported teleophthalmology's potential impacts and benefits with these lived experiences within the HERMES study.
- This study collected data from multiple stakeholders, including primary and secondary eye care professionals and patients.
- A limitation of this study was that all ophthalmologists interviewed were involved in the HERMES study and, therefore may have had more knowledge of teleophthalmology than others.

Introduction

Primary eyecare in the U.K. is mainly delivered by community optometry practices [1], [2]. If patients are suspected of having a retinal condition, referrals to hospital eye services (HES) are typically processed by their general practitioner (G.P.) based on recommendations from the community-based optometrist. Thus, optometrists are often not involved in making direct referrals using electronic referral platforms or informed of outcomes. The additional step can reduce the specificity of clinical details included in the referral, as GPs are not specialists in eye care and rarely have the time or expertise to undertake eye examinations [3]. For several reasons, including concerns over capacity, changes in practice due to the Covid-19 pandemic, and the high number of referrals to HES, there is a need for disruptive changes in the optometric referral pathways for suspected retinal conditions (SRC)[4]. We explore the experiences of patients, optometrists, and ophthalmologists of teleophthalmology for SRC.

For peer review only

Teleophthalmology describes the process of providing health information with medical technology at a distance, geographical, temporal or both [5], [6], to facilitate decision-making. In our study, we focused on an asynchronous platform; this allowed through uploading clinical information and multi-modal retinal imaging, i.e., fundus photography and macular OCT scans directly from the optometry practice, for later review by the receiving HES-based ophthalmologists. A benefit of the custom-built study platform, when compared to recent attempts for the commissioning of teleophthalmology-like store-and-forward referral pathways, is that it enables optometrists to upload full-volume optical coherence tomography (OCT) scans from multiple device manufacturers, in both their proprietary and open-source file format, which can then be parsed and reconstructed into a high-quality full volume scan, directly viewable on the platform's embedded viewer [7], [8]. Given the critical role of OCT imaging for diagnosing and managing medical retina conditions, a minimum clinical dataset including full-volume OCT is a prerequisite for the safe and efficient delivery of teleophthalmology referral triaging, providing ophthalmologists with essential information to confidently make referral triaging decisions. In the absence of teleophthalmology, or when attempts to implement teleophthalmology-like pathways are made with minimal input from clinical informatics and HES-based clinician and imaging experts, triaging ophthalmologists receive referrals, either without any accompanying imaging or frequently with one or a few selected cross-sectional images, not the entire volume [9], then referred patients are seen face to face to assess whether further investigation or treatment is needed.

The non-urgent referral pathway with and without teleophthalmology is presented in Figure 1. A recent systematic review reported that teleophthalmology and digital referrals could reduce waiting time, costs, and unnecessary referrals. It also noted that teleophthalmology could lead to earlier detection and diagnosis [10] and as such is an underutilised resource for HES. This review was based on reviewing referrals, not people's first-hand experiences. Therefore, there is a need to understand how implementation would affect users in practice.

Reports have shown that the growing use of OCT scanning has increased the number of referrals to HES [11], [12]; therefore, teleophthalmology has been suggested to reduce unnecessary referrals, manage growing capacity concerns and potentially manage increasing workloads by reviewing referrals before patients are seen in clinics.

We present findings from a study linked with a cluster randomised clinical trial (HERMES) evaluating the effectiveness of a teleophthalmology platform [1]. Those in the intervention arm of the trial were using teleophthalmology to refer patients to HES, while those in the control arm were using their regular referral pathways. We report the novel qualitative findings of patients' and healthcare professionals' experiences to understand the practical implications of implementing teleophthalmology.

Materials and Method

This study's overall aims, objectives and recruitment strategy are detailed elsewhere [13]; we summarise key points here. We undertook semi-structured interviews with 41 participants from across the UK, between December 2021 and May 2022. All participants were recruited from sites participating in the wider HERMES trial.These comprised 6 ophthalmologists who were all making remote referral decisions; 18 community optometrists, of whom 12 were from the intervention arm and 6 from the control arm of the HERMES study; and 17 patients recruited from community practices, of which 14 were from clinics in the intervention arm and 3 from clinics in the control arm. These sites were affiliated with four NHS Trusts; Moorfields Eye Hospital NHS Foundation Trust, Central Middlesex Hospital at London North West University Healthcare NHS Trust, Birmingham University Hospital NHS Foundation Trust and North West Anglia NHS Foundation Trust. A semi-structured topic guide (attached in Supplementary Material) was used for all interviews and was tailored to each participant group. All interviews were audio-recorded with consent. Five interviews were manually transcribed verbatim to aid familiarisation; the remaining interviews were transcribed using Scrintal Software. All transcripts were anonymised and checked for accuracy. The transcripts were coded using NVivo by an independent researcher who did not conduct the interviews, using inductive thematic analysis methods [14], [15]. 35 codes were initially defined and discussed with the research team. These were then refined and categorised into overarching themes. We present the three main themes which explore experiences of teleophthalmology for referrals for SRC.

For peer review only - http://bmjopen.bmj.com/site/about/guidelines.xhtml

Patients and Public Involvement

Eighteen patients were consulted in the planning and development of the wider HERMES Study, which has been detailed in the protocol paper [13]. The insights raised about understanding the benefits of teleophthalmology contributed to the design of this qualitative study. As noted above, 17 patients participated in interviews.

Results

We present our findings under three themes, *Efficiencies of Teleophthalmology; Teleophthalmology enables Feedback* and *Concerns about Teleophthalmology.* We found most participants were optimistic about the implementation of teleophthalmology in the optometric referral pathway due to the efficiencies the platform would enable. All participants expressed needing feedback during the referral process to improve care and highlighted some concerns.

Efficiencies of Teleophthalmology

All welcomed Teleophthalmology due to its ability to improve patient and clinician experiences. There is regional variation in referral pathways depending on the specific condition; GPs typically process routine referrals for SRC. However, it was reported that GPs were not always suited to create referrals due to their limited skillset in specialist eyecare. Patients often described GPs as the unnecessary "middleman". Patients reported being keen to be referred directly by their optometrist and felt this would reduce their waiting time to hear back from HES if referrals were processed directly.

"If the optician can do the referral directly rather than you know, the optician telling me you'll need to go and see your GP who will refer you (...) I would be more comfortable because they [optometrist] know what they're doing whereas the GP is just saying you are alright then, if your optician told you that, then I'll send you." (Patient 11)

In the HERMES study, optometrists used teleophthalmology to refer patients to HES, enabling a quicker referral process direct to triaging ophthalmologists. They shared that teleophthalmology could improve patient satisfaction and help relieve hospital capacity pressures by reducing unnecessary hospital visits.

"I think the whole teleophthalmology thing will improve patient satisfaction and it makes life a lot easier, less patients, elderly patients having to find transport to the hospital, and being dilated once in the optometry practice and then being dilated again, back at the hospital and patient transport having to be arranged, so overall good, big saving of cost and finance and less crowded waiting rooms at the hospital." (Optometrist 18)

Ophthalmologists shared that the teleophthalmology platform introduced uniformity to the referral process by requiring the same data fields to be completed for each patient, which was easy for the referring optometrist. This enabled referrals to be triaged and reviewed more quickly: referral decisions could be made promptly, and the appropriate triaging decision could be made regarding the indication and the urgency for a hospital visit.

"It's more informative because, as you know, the platform has the questions with the tick box, um, on the optician findings which [is] not always involved in a classic referral proforma. And obviously, it has the imaging as well, which helps us to make a decision very quickly." (Ophthalmologist 1)

As mentioned, the key benefit of the teleophthalmology platform is the ability to review and triage patient referrals without them having to attend a face-to-face appointment. All participants recognised and shared the advantage of saving time and resources by using teleophthalmology.

Teleophthalmology Enables Feedback

Some patients reported that they were happy for teleophthalmology to be used to assess their referrals as they would not want to attend HES if not required. Still, they expected feedback to explain the reason for not being seen for a

For peer review only

face-to-face appointment. This was not always provided. Some patients reported dissatisfaction with their referral experience due to the lack of communication. This was also shared in the context of not receiving feedback promptly. Patients expected to hear about their referral decision more promptly through the teleophthalmology process, which increased concerns over their eye health when this expectation was not satisfied. In these cases, patients wanted to be seen or told directly and promptly why an appointment was not required and not to be kept waiting in uncertainty. Teleophthalmology can overcome this expectation discrepancy through accurate information presentation and timely and clear feedback.

"If the patients were to get a letter or some form of communication from the hospital or the specialist to reassure them that your case has been looked at and this is what has been concluded, I think that would be enough to put somebody's mind at ease." (Patient 10)

Optometrists stated that one of the significant benefits of teleophthalmology was the ability to receive feedback from ophthalmologists. Optometrists reported that when patients were referred to HES in the absence of teleophthalmology, patients would often return to them seeking more information and advice about their care; therefore, it was important for optometrists to be involved in the referral pathway and remain informed of their patients' management plans. Optometrists shared that many patients were not a reliable source of information about their eye health/treatment, which could affect future care or monitoring they provided.

"Once we refer the patient, we don't actually know then what is happening thereafter, unless we chase the patient or, our patients are quite loyal, so they would, we would see them a year later, we will say or remember, we referred you last time, what happened? (…) So, it's actually we're basing it of what the patient is then telling us, so we are actually getting like the second story through the patient rather than the actual clinical information." (Optometrist 12)

By receiving feedback, optometrists can also verify whether their referrals were appropriate and audit themselves to improve the quality of their referrals.

"Because if we keep referring something that we think is urgent, but [the] ophthalmologist tells us this is not urgent, and if you learn by that, that's going to help you, you see. Right now, there's no feedback. (…). But if I got feedback from the ophthalmologist that saw the patient and I will know for next time when I see that similar sort of situation that well, actually this isn't urgent." (Optometrist 10)

Having a system where the community-based clinic is connected to the HES was also seen as a great benefit for ophthalmologists. They welcomed being able to provide feedback to the referring optometrists, especially to enable the sharing of referral decisions directly and concurred with the need to provide feedback on the referral quality to improve future referrals. It was suggested that the teleophthalmology system should send referral replies to the referring optometrist, patient, and GP so all are informed of the outcome.

Concerns about Teleophthalmology

There were some concerns about using technology to manage patient referrals. Some patients still wanted the reassurance of seeing a clinician rather than having their referral decision and notification completed remotely. Seeing someone face-to-face provided the holistic care some patients reported wanting and addressed their worries and anxieties.

"I think [if] you don't get a chance to see the patient yourself, there is something about looking at data visually transactionally, that is fine, but there is also something about talking to the patients about how they're feeling and how they're coping with things." (Patient 10)

Optometrists were mainly concerned with the practicalities of implementing a new system into their workflow. This included concerns over training to use the equipment, the reliability of network connectivity and equipment costs

For peer review only - http://bmjopen.bmj.com/site/about/guidelines.xhtml

that some smaller practices may not be able to bear, as well as remuneration for their time for taking on additional roles. Some also reported that completing a referral on the teleophthalmology platform took time.

"The barriers would be cost, because this is all based on the information that is being sent from an OCT device, yeah, as part of the process of referral, it's not just from a letter, so when it comes to having the equipment, that's an immediate barrier. And having the right remuneration for the equipment." (Optometrist 2)

Ophthalmologists also shared these concerns; however, they were positive towards the ability of teleophthalmology, enabling them to use their time more efficiently.

Discussion

Whilst previous work has focused on the efficacy and efficiency of teleophthalmology platforms through reviewing referrals [12], [16], we report insights based on experiences from patients, optometrists, and ophthalmologists to validate previous findings on perceptions of using teleophthalmology for suspected retinal conditions.

All participants recognised the value of implementing a teleophthalmology system into their ophthalmic care pathway due to its potential to improve patient care and health services efficiencies. This is supported by others [17], who found through a review of referrals that teleophthalmology can reduce unnecessary HES visits and significantly impact patient anxieties [18]. Our study has shown this in practice, with many patients sharing that they would not want to attend HES if not required.

Both optometrists and ophthalmologists reported that teleophthalmology's significant advantage is the ability to electronically refer patients directly from optometrist practices to hospital eye services, which can significantly reduce the waiting time for patients. The ability to triage referrals electronically also enabled ophthalmologists to provide replies and feedback to the referring optometrist via the teleophthalmology platform, which they greatly valued.

Implementing teleophthalmology into the eye care pathway would remove the burden on GPs of having to process patient referrals, but they must be informed of such referrals. GPs have, in principle, supported the suggestion of optometrists referring patients directly [16], [19], and we found that patients and optometrists would support this change in practice. There is great value in involving optometrists in the referral process, as it has been reported that this could reduce unnecessary referrals by approximately half [12]

The overarching theme shared by all participants that substantiates many of the benefits of the teleophthalmology platform is the potential of the platform to facilitate the provision of feedback. The importance of receiving timely feedback in eye care, in general, has been reported by others [3], [20] and was essential for patients to alleviate their concerns over their eye health. Harvey et al. (2022) specifically outlines the factors that could affect the provision of feedback, and a key implication of their work is the call for technology to support this provision. Thus, we found that teleophthalmology could overcome concerns in the optometric referral pathway.

Whilst feedback can keep both patients and optometrists informed [21], it can also improve future referral quality through open conversations between referring clinicians. Our results concur as we report optometrists greatly value receiving referral replies directly from ophthalmologists to remain informed of their patients' care and audit their referrals. Additionally, previous research has highlighted that the lack of communication between optometrists and ophthalmologists can be problematic [22], [23]; therefore, implementing a teleophthalmology platform could help to overcome this.

Potential barriers raised by some optometrists were the initial setup costs, which include time, training, and financial costs. Whilst others have reported this should be considered [10], [16], we found this to be a key concern in practice. According to the current General Ophthalmic Services contract (2023), OCT scans are not a contracted service in optometric care [24]. Therefore, there is a need to ensure optometrists are appropriately remunerated for providing this service. Optometrists would also need to have appropriate access to NHS e-referral systems to expediently refer patients to the HES system. Others have begun to explore the cost-effectiveness of implementing teleophthalmology

For peer review only

systems [25]; further work is needed to establish health-economic benefits concerning SRC that were raised in our study.

To successfully implement teleophthalmology into the optometric referral pathway, there needs to be an investment to enable parity amongst optometry practices to support new technology setup. Whilst the implementation of teleophthalmology was perceived to initially increase the workload of optometrists to process referrals and ophthalmologists to triage referrals, with the correct remuneration, there is significant potential to relieve pressures on stretched eye care services.

We acknowledge study limitations including the influence of participation bias. The participants who chose to participate in our research may have different views from those who did not participate, which have not been captured in our study. We also recognise that the ophthalmologists who chose to participate were involved in the HERMES study itself, which led to increased knowledge of teleophthalmology. Future work should endeavour to recruit a more diverse sample of participants to capture broader views on the experiences of teleophthalmology. However, the strengths of our study include the use of in-depth interviews, through which, we were able to elicit the lived experiences of those involved in the study, many of whom had direct experience with the teleophthalmology platform. We were also able to recruit participants across the stakeholder group, thus providing multiple perspectives on the teleophthalmology pathway. Optometrists and ophthalmologists provided their perspectives on using the platform and the practicalities involved in use, whilst patients reported on the personal impacts of navigating their eye-health journey through teleophthalmology. These diverse perspectives have enabled us to corroborate and extend existing understanding of the practical implications of implementing teleophthalmology.

Conclusions

Implementing teleophthalmology into the optometric referral pathway has numerous benefits, as outlined by all participants. Through our in-depth interview study with patients, optometrists, and ophthalmologists, we found that they generally report great value in implementing teleophthalmology through improving efficiency and the ability to provide and receive feedback. Patients were satisfied if their referrals were reviewed with teleophthalmology to reduce the possibility of unnecessary HES visits if this was clearly and efficiently communicated back to them. Optometrists felt they were better suited than GPs to write and process patient referrals and would feel more valued if they were more directly involved in the pathway. Finally, ophthalmologists were pleased with a system enabling them to manage their caseloads more efficiently. Further efficiencies teleophthalmology can promote include removing the burden on GPs, the time patients wait to be seen by HES, the time it takes for ophthalmologists to review and provide referral replies and finally, the overarching benefit to all participants of being involved and receiving feedback. Future work could explore how to overcome barriers such as connectivity and the specific health economics of implementing teleophthalmology to validate our findings.

Acknowledgements

We thank all those involved in this research, including participants and the wider research team.

Funding Statement

This work was funded by NIHR Health Technology Assessment grant number 18/182.

Competing Interests

We have no competing interests to declare.

Author Contributions

DP analysed the data and prepared the manuscript. SA, KB & AB designed the study protocol, SA undertook recruitment and data collection. SA, JC & AB contributed analysis as presented by DP. All authors reviewed and contributed to subsequent drafts and approved the final manuscript.

For peer review only

Ethics Statement

Ethical approvals were gained from London-Bromley Research Ethics Committee (REC 20/LO/1299). Informed consent was obtained from participants after details of the study, including the aims and methods, were disclosed.

Data Availability Statement

We are happy to be contacted to share anonymised data. Please contact the corresponding or senior author for requests.

References

[1] J. E. D. Han and others, 'Teleophthalmology-enabled and artificial intelligence-ready referral pathway for community optometry referrals of retinal disease (HERMES): a Cluster Randomised Superiority Trial with a linked Diagnostic Accuracy Study-HERMES study report 1-study protocol', *BMJ Open*, vol. 12, no. 2, p. e055845, 2022.
[2] B. Bowling, S. D. M. Chen, and J. F. Salmon, 'Outcomes of referrals by community optometrists to a hospital glaucoma service', *Br J Ophthalmol*, vol. 89, no. 9, pp. 1102–1104, Sep. 2005, doi: 10.1136/bjo.2004.064378.
[3] T. Pierscionek, J. Moore, and B. Pierscionek, 'Referrals to ophthalmology: optometric and general practice comparison', *Ophthalmic and Physiological Optics*, vol. 29, no. 1, pp. 32–40, 2009.
[4] Y. C. Tham and others, 'New digital models of care in ophthalmology, during and beyond the COVID-19 pandemic', *Br J Ophthalmol*, vol. 106, no. 4, pp. 452–457, 2022.
[5] L. J. Caffery and others, 'Models of care in tele-ophthalmology: A scoping review', *J Telemed Telecare*, vol. 25, no. 2, pp. 106–122, 2019.
[6] T. S. Surendran and R. Raman, 'Teleophthalmology in Diabetic Retinopathy', *J Diabetes Sci Technol*, vol. 8, no. 2, pp. 262–266, 2014.
[7] 'Improve referrals to eye care services', NHS Transformation Directorate. Accessed: Dec. 09, 2023. [Online]. Available: https://transform.england.nhs.uk/key-tools-and-info/digital-playbooks/eye-care-digital-playbook/improve-referrals-to-eye-care-services/
[8] C. Roberts, 'How digital eye care referrals will transform care'. 2021.
[9] H. a. S. C. Committee, 'Evaluation of Government commitments made on the digitisation of the NHS. Fourth Special Report of Session 2022-23', 2023.
[10] M. P. Conway and others, 'Investigating the Role of Optometrists in Teleophthalmology and the Implications of Increasing Access to Advanced Imaging Techniques and Digital Referral: A Systematic Search and Review', *Telemedicine and e-Health*, vol. 27, no. 9, pp. 974–981, 2021.
[11] J. X. Lee, V. Manjunath, and S. J. Talks, 'Expanding the role of medical retina virtual clinics using multimodal ultra-widefield and optical coherence tomography imaging', *Clin Ophthalmol*, vol. 12, pp. 2337–2345, 2018.
[12] C. Kern and others, 'Implementation of a cloud-based referral platform in ophthalmology: making telemedicine services a reality in eye care', *Br J Ophthalmol*, vol. 104, no. 3, pp. 312–317, 2020.
[13] A. Blandford and others, 'Protocol for a qualitative study to explore acceptability, barriers and facilitators of the implementation of new teleophthalmology technologies between community optometry practices and hospital eye services', *BMJ Open*, vol. 12, no. 7, p. e060810, 2022.
[14] V. Braun and V. Clarke, 'Using Thematic Analysis in Psychology', *Vol. 3*, pp. 77–101, 2006.
[15] V. Braun and V. Clarke, *Successful qualitative research: A practical guide for beginners*. Sage, 2013.
[16] S. P. Kelly and others, 'Teleophthalmology with optical coherence tomography imaging in community optometry. Evaluation of a quality improvement for macular patients', *Clinical Ophthalmology*, vol. 5, p. 1673, 2011.
[17] D. V. Muttuvelu and others, 'Danish teleophthalmology platform reduces optometry referrals into the national eye care system', *BMJ Open Ophthalmology*, vol. 6, no. 1, p. e000671, 2021.
[18] C. J. Davey, C. Harley, and D. B. Elliott, 'Levels of state and trait anxiety in patients referred to ophthalmology by primary care clinicians: a cross-sectional study', *PLoS One*, vol. 8, no. 6, p. e65708, 2013.
[19] NHS, '2023/24 priorities and operational planning guidance', Jan. 2023. Accessed: Dec. 09, 2023. [Online]. Available: https://www.england.nhs.uk/wp-content/uploads/2022/12/PRN00021-23-24-priorities-and-operational-planning-guidance-v1.1.pdf

For peer review only

[20] K. Harvey and others, ‘Referrals from community optometrists in England and their replies: A mixed methods study’, *Ophthalmic and Physiological Optics*, vol. 42, no. 3, pp. 454–470, 2022.
[21] D. M. Baldwin and others, ‘Patient preferences for notification of normal laboratory test results: a report from the ASIPS Collaborative’, *BMC family practice*, vol. 6, no. 1, pp. 1–7, 2005.
[22] A. S. O�Malley and J. D. Reschovsky, ‘Referral and consultation communication between primary care and specialist physicians: finding common ground’, *Archives of internal medicine*, vol. 171, no. 1, pp. 56–65, 2011.
[23] D. Townsend and others, ‘Health professionals� and service users� perspectives of shared care for monitoring wet age-related macular degeneration: a qualitative study alongside the ECHoES trial’, *BMJ open*, vol. 5, no. 4, p. e007400, 2015.
[24] N. England, ‘General Ophthalmic Mandatory Services Model Contract’, NHS, Sep. 2023. Accessed: Dec. 09, 2023. [Online]. Available: https://www.england.nhs.uk/wp-content/uploads/2018/08/PRN00242-General-ophthalmic-mandatory-services-model-contract-September-2023.pdf
[25] N. Sharafeldin and others, ‘Review of economic evaluations of teleophthalmology as a screening strategy for chronic eye disease in adults’, *Br J Ophthalmol*, vol. 102, no. 11, pp. 1485–1491, 2018.

List of Figures

Figure 1: The non-urgent patient referral pathway with and without teleophthalmology.

For peer review only

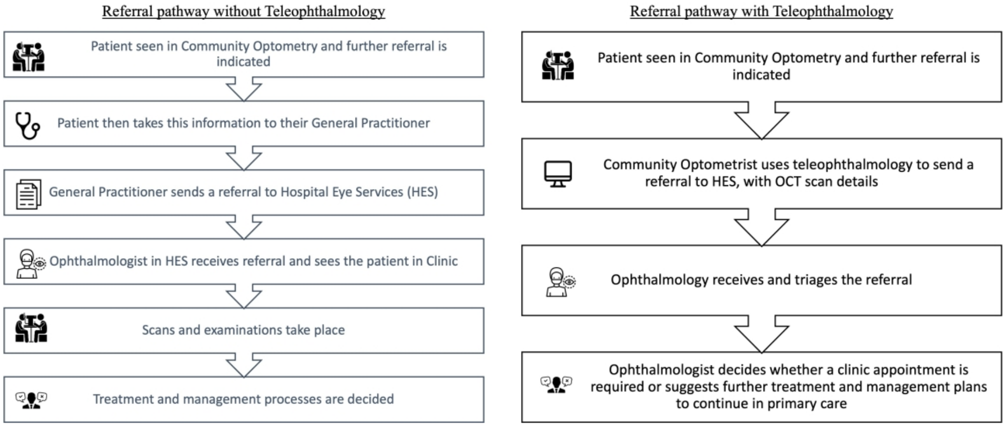

317x135mm (330 x 330 DPI)

Interview topic guide- Patients

1. Information and consent

- Briefly re-introduce the topic, the purpose of the overall project and the specific aim of the HCI study
- Provide assurances regarding confidentiality and how information will be processed
- Check willingness to continue to participate
- Obtain informed consent (if not taken in advance) and permission to be audio/video recorded

2. Introduction

Thank you for taking part in this interview. We really appreciate you taking the time to talk with us. As I explained earlier, we are keen to talk to you about the use of new tele-eye care technologies in the referral process between community optometric practices and hospital eye services. We want to hear your views on these technologies and whether they would work for you in either a good or bad way. There are no right or wrong answers, so feel free to tell us what you think, and if there is something that needs to be improved.

The interview is likely to take around 30 minutes, you are free to stop and take a break whenever you feel like. It is also voluntary, which means you can opt out at any time for any reason.

3. Background information

- So, first of all: I would like to know more about you and your use of primary and secondary eye services.
- How old are you?
- Do you have any known eye problems?
- How often do you visit your optician?
    - Do you do regular check-ups, or do you visit the practice when you have eye problems only?
    - When was your last visit to optometric practice [i.e., the one before your most recent appointment)?

For peer review only

- What led to you to making your most recent eye appointment [i.e., the one that has led to the referral]?
- What did you expect to happen during that particular appointment?

- Have you been referred to hospital for eye care before?

4. Questions related to the teleophthalmology platform

- Can you describe to me your most recent visit to the optometric practice services? [i.e., the one you just had with the OCT scan?]
  - Can you describe to me the steps that you went through during the consultation with the optometrist?
  - **For the intervention arm:**
    - Have you noticed any difference between the care you received this time and last time? Were you asked to do something different during this consultation to what you were used to do? What was it? What are your thoughts on this?
  - Were the OCT scan results discussed with you? When were you informed about the referral decision?
  - What were you told will happen next? How did you feel about that?
  - How long did you tell you it will take to see a doctor?
- **For the intervention arm**
- Have you **received feedback** about your referral?
  - How long did it take from the time you were referred by your optician to the time you received the feedback (referral letter)? What do you think about this waiting time?
  - What were the information included in the feedback letter? (do you remember?), what do you think about that?
  - What happened next?
- **For the control arm**
  - What do you think a tele-eye care service or a teleophthalmology service in the referral process is? **Explain what teleophthalmology is in the context of the HERMES project.**
- Generally, what do you think about this new referral process?
- What do you think the benefits of this service are?
- What do you think the problems with this service are?

For peer review only

- What do you think the disadvantages would be for you as a patient if this type of service was implemented in real life?
- In this type of service, an optician at the community optometry practice and an ophthalmologist at the hospital will be involved in the referral process, who would you be most confident in their decision? And why?
- How would you feel if you were referred to hospital for eye care when it was not necessary?
  - How would you feel spending this time on a visit that wasn't necessary?
  - What do you think should be done to avoid referring you when it is not necessary?
- Some of the opticians refer all cases with suspected retinal conditions to hospital, which may include those who are urgent and those that turn out to be unnecessary (unnecessary referrals). So when hospital receive these referrals, the hospital has two options- **Option 1:** See all patients referred by opticians and provide re-assurances to everyone regarding their eye conditions or **Option 2:** hospital sees mainly urgent cases who require hospital care.
  - What do you think of these options? Which one would you prefer? Why?

6. End of the interview

We have reached the end of the interview. Would you like to share any more ideas or thoughts?

Thank you so much for taking the time to participate in the study, we will use this information to inform the design of a future tele-eye care service.

For peer review only

Interview topic guide – Primary care professionals - Control

1. Information and consent

- Briefly re-introduce the topic, the purpose of the overall project and the specific aim of the HCI study
- Obtain informed consent (if not taken in advance) and permission to be audio/video recorded

2. Introduction

Thank you for taking part in this interview. We really appreciate you taking the time to talk with us. As I explained earlier, we are keen to talk to you about the use of new teleophthalmology technologies in the referral process between community optometry practices and hospital eye services. We want to hear your views on these technologies, and what factors you think may influence their implementation in real life**.** There are no right or wrong answers, so feel free to tell us what you think, and if there is something that needs to be improved.

The interview is likely to take around 30-45 minutes. Your participation is voluntary, and you can withdraw from the study at any time.

3. Background Information

- First of all: I will start by asking you a few questions to know more about you.
- What is your professional background?
- How long have you been working in community optometry practices?
- How often do you refer patients with retinal conditions to hospital eye services? (in a week what is the average number of cases identified with retinal conditions)
- Can you explain to me the different referral pathways that you follow when you identify someone with a suspected retinal condition (usual not through the teleophthalmology platform)
- What do you think of the referral process?
    - What are the issues or barriers that you face when referring patients?
- How do you think the referral process can be improved?

4. Teleophthalmology platform

4.1 Expectation/coherence

- What do you think a teleophthalmology platform is? How different is it from the traditional system?
- What would you like to see in a teleophthalmology platform?
- How do you think a teleophthalmology platform would benefit/improve the referral process for eye conditions?

For peer review only

- What barriers do you think there will be to using a teleophthalmology platform in the context of referrals?
- What do you think would need to change to incorporate a teleophthalmology platform in your practice?

6. End of the interview

We have reached the end of the interview. Would you like to share any more ideas or thoughts?

Thank you so much for taking the time to participate in the study, we will use this information to inform the design of future teleophthalmology services.

Interview topic guide – Primary care professionals - Intervention

1. Information and consent

- Briefly re-introduce the topic, the purpose of the overall project and the specific aim of the HCI study
- Obtain informed consent (if not taken in advance) and permission to be audio/video recorded

2. Introduction

Thank you for taking part in this interview. We really appreciate you taking the time to talk with us. As I explained earlier, we are keen to talk to you about the use of new teleophthalmology technologies in the referral process between community optometry practices and hospital eye services. We want to hear your views on these technologies, and what factors you think may influence their implementation in real life. There are no right or wrong answers, so feel free to tell us what you think, and if there is something that needs to be improved.

For peer review only

The interview is likely to take around 30-45 minutes. Your participation is voluntary, and you can withdraw from the study at any time.

3. Background Information

- First of all: I will start by asking you a few questions to know more about you.
- What is your professional background?
- How long have you been working in community optometry practices?
- How often do you refer patients with retinal conditions to hospital eye services? (in a week what is the average number of cases identified with retinal conditions)
- Can you explain to me the different referral pathways that you follow when you identify someone with a suspected retinal condition (usual not through the teleophthalmology platform)
- What do you think of the referral process?
    - What are the issues or barriers that you face when referring patients?
- How do you think the referral process can be improved?

4. Teleophthalmology platform

4.1 Experience/cognitive participation

- How long have you been using this new teleophthalmology platform?
- Can you describe to me the steps you follow when you refer a patient with a suspected retinal condition through the new teleophthalmology platform?
- How long does it take you to do a referral on this platform? What do you think of that?
- What did you like about this new platform? Why?
- What did you not like about this new platform? Why?
    - What were the issues that you faced when using the platform?
    - Do you have any concerns regarding the use of this platform?
- What type of support did you receive to use this platform (e.g., training, support staff)?

4.2 Experience/collective action

- What changes did you have to make in your work routine to incorporate this new technology?
- How will this new platform fit in your current practice?
- How do you think this new platform will impact/improve your practice, patient care and your working relationship with secondary care?

For peer review only

4.3 Reflective monitoring

- What do you think will be the benefits of this new teleophthalmology platform on the referral process, both short term and long term?
  - How do you think this new teleophthalmology platform can help reduce unnecessary referrals?
  - How do you think this new teleophthalmology platform can help improve the quality of referrals?
- What barriers/disadvantages do you think there will be to using this new platform on the referral process?
- What do you think would need to change for this technology to be successfully embedded in routine eye practice (e.g., training…)?

6. End of the interview

We have reached the end of the interview. Would you like to share any more ideas or thoughts?

Thank you so much for taking the time to participate in the study, we will use this information to inform the design of future teleophthalmology services.

Questions for ophthalmologists:

- I will be asking you a few questions regarding the use of the new teleophthalmology platform in the referral process.
- I will audio record the session, are you happy for me to do so? Consent form points.
  1. Can you sketch out the overall referral process for retinal conditions and how it works from your trust's perspective?
  2. What are the main issues you face in the referral process from community optometry practices?
  3. About how many new retinal referrals do you deal with per week? Usually how do you organize this aspect of your work? ** Usual practice**
  4. For the HERMES platform, how are you notified about new referrals on the system? How often do you check the system for new referrals, or how soon do you review the referral? How many referrals do you receive in this system per week?
  5. How long does it take you to review each referral? What do you think of this duration? Reasonable, good, long, can be improved?
  6. Who else check the referrals on the teleophthalmology platform at your trust? How do you coordinate your work with them? Do you distribute the referrals amongst

For peer review only

yourselves, or do you review each new referral? How do you validate your referral decision?

7. So far, what do you think about the quality of the referrals received by this platform? Have you observed any changes compared to usual route?
8. What are the things that you liked about the platform, things that are working well so far?
9. What are the pain points of the platform? What are the things that you did not like? And how can they be improved?
10. What happens next, after you decided on the referral?
11. Looking to the future, how do you envision that this system will be rolled out across your trust or are a couple of consultants always going to be able to assess all OCT scans?
12. What do you think would need to change for this technology to be successfully embedded in routine eye practice?

For peer review only

Standards for Reporting Qualitative Research (SRQR)*

http://www.equator-network.org/reporting-guidelines/srqr/

Title and abstract

	Page/line no(s).
**Title** - Concise description of the nature and topic of the study Identifying the study as qualitative or indicating the approach (e.g., ethnography, grounded theory) or data collection methods (e.g., interview, focus group) is recommended	1
**Abstract** - Summary of key elements of the study using the abstract format of the intended publication; typically includes background, purpose, methods, results, and conclusions	1

Introduction

**Problem formulation** - Description and significance of the problem/phenomenon studied; review of relevant theory and empirical work; problem statement	1-2
**Purpose or research question** - Purpose of the study and specific objectives or questions	1-2

Methods

**Qualitative approach and research paradigm** - Qualitative approach (e.g., ethnography, grounded theory, case study, phenomenology, narrative research) and guiding theory if appropriate; identifying the research paradigm (e.g., postpositivist, constructivist/ interpretivist) is also recommended; rationale**	2
**Researcher characteristics and reflexivity** - Researchers' characteristics that may influence the research, including personal attributes, qualifications/experience, relationship with participants, assumptions, and/or presuppositions; potential or actual interaction between researchers' characteristics and the research questions, approach, methods, results, and/or transferability	/
**Context** - Setting/site and salient contextual factors; rationale**	2
**Sampling strategy** - How and why research participants, documents, or events were selected; criteria for deciding when no further sampling was necessary (e.g., sampling saturation); rationale**	2
**Ethical issues pertaining to human subjects** - Documentation of approval by an appropriate ethics review board and participant consent, or explanation for lack thereof; other confidentiality and data security issues	2
**Data collection methods** - Types of data collected; details of data collection procedures including (as appropriate) start and stop dates of data collection and analysis, iterative process, triangulation of sources/methods, and modification of procedures in response to evolving study findings; rationale**	2

For peer review only - http://bmjopen.bmj.com/site/about/guidelines.xhtml

**Data collection instruments and technologies** - Description of instruments (e.g., interview guides, questionnaires) and devices (e.g., audio recorders) used for data collection; if/how the instrument(s) changed over the course of the study	2
**Units of study** - Number and relevant characteristics of participants, documents, or events included in the study; level of participation (could be reported in results)	2
**Data processing** - Methods for processing data prior to and during analysis, including transcription, data entry, data management and security, verification of data integrity, data coding, and anonymization/de-identification of excerpts	2
**Data analysis** - Process by which inferences, themes, etc., were identified and developed, including the researchers involved in data analysis; usually references a specific paradigm or approach; rationale**	2
**Techniques to enhance trustworthiness** - Techniques to enhance trustworthiness and credibility of data analysis (e.g., member checking, audit trail, triangulation); rationale**	/

Results/findings

**Synthesis and interpretation** - Main findings (e.g., interpretations, inferences, and themes); might include development of a theory or model, or integration with prior research or theory	2-4
**Links to empirical data** - Evidence (e.g., quotes, field notes, text excerpts, photographs) to substantiate analytic findings	2-4

Discussion

**Integration with prior work, implications, transferability, and contribution(s) to the field** - Short summary of main findings; explanation of how findings and conclusions connect to, support, elaborate on, or challenge conclusions of earlier scholarship; discussion of scope of application/generalizability; identification of unique contribution(s) to scholarship in a discipline or field	4-5
**Limitations** - Trustworthiness and limitations of findings	5

Other

**Conflicts of interest** - Potential sources of influence or perceived influence on study conduct and conclusions; how these were managed	6
**Funding** - Sources of funding and other support; role of funders in data collection, interpretation, and reporting	6

*The authors created the SRQR by searching the literature to identify guidelines, reporting standards, and critical appraisal criteria for qualitative research; reviewing the reference lists of retrieved sources; and contacting experts to gain feedback. The SRQR aims to improve the transparency of all aspects of qualitative research by providing clear standards for reporting qualitative research.

For peer review only - http://bmjopen.bmj.com/site/about/guidelines.xhtml

For peer review only

**The rationale should briefly discuss the justification for choosing that theory, approach, method, or technique rather than other options available, the assumptions and limitations implicit in those choices, and how those choices influence study conclusions and transferability. As appropriate, the rationale for several items might be discussed together.

Reference:

O'Brien BC, Harris IB, Beckman TJ, Reed DA, Cook DA. **Standards for reporting qualitative research: a synthesis of recommendations.** *Academic Medicine*, Vol. 89, No. 9 / Sept 2014 DOI: 10.1097/ACM.0000000000000388

For peer review only

For peer review only - http://bmjopen.bmj.com/site/about/guidelines.xhtml
